# The importance of evaluating specific myeloid malignancies in epidemiological studies of environmental carcinogens

**DOI:** 10.1186/s12885-021-07908-3

**Published:** 2021-03-06

**Authors:** K. A. Mundt, L. D. Dell, P. Boffetta, E. M. Beckett, H. N. Lynch, V. J. Desai, C. K. Lin, W. J. Thompson

**Affiliations:** 1 Cardno ChemRisk, Boston, MA USA; 2 Ramboll US Consulting Inc., Amherst, MA USA; 3 Stony Brook Cancer Center, Stony Brook, NY USA; 4 Department of Medical and Surgical Sciences, University of Bologna, Bologna, Italy; 5 Mount Sinai Hospital, New York, NY USA

**Keywords:** Myeloid, AML, MDS, CML, MPN, Epidemiology, Benzene, Formaldehyde, 1,3-butadiene, Tobacco smoking

## Abstract

**Introduction:**

Although myelodysplastic syndrome (MDS), acute myeloid leukemia (AML), myeloproliferative neoplasms (MPN) – including chronic myeloid leukemia (CML) – and myelodysplastic/myeloproliferative neoplasms (MDS/MPN) are largely clinically distinct myeloid malignancies, epidemiological studies rarely examine them separately and often combine them with lymphoid malignancies, limiting possible etiological interpretations for specific myeloid malignancies.

**Methods:**

We systematically evaluated the epidemiological literature on the four chemical agents (1,3-butadiene, formaldehyde, benzene, and tobacco smoking, excluding pharmaceutical, microbial and radioactive agents, and pesticides) classified by the International Agency for Research on Cancer as having sufficient epidemiological evidence to conclude that each causes “myeloid malignancies.” Literature searches of IARC Monographs and PubMed identified 85 studies that we critically assessed, and for appropriate subsets, summarized results using meta-analysis.

**Results:**

Only two epidemiological studies on 1,3-butadiene were identified, but reported findings were inadequate to evaluate specific myeloid malignancies. Studies on formaldehyde reported results for AML and CML – and not for MDS or MPN – but reported no increased risks. For benzene, several specific myeloid malignancies were evaluated, with consistent associations reported with AML and MDS and mixed results for CML. Studies of tobacco smoking examined all major myeloid malignancies, demonstrating consistent relationships with AML, MDS and MPN, but not with CML.

**Conclusions:**

Surprisingly few epidemiological studies present results for specific myeloid malignancies, and those identified were inconsistent across studies of the same exposure, as well as across chemical agents. This exercise illustrates that even for agents classified as having sufficient evidence of causing “myeloid malignancies,” the epidemiological evidence for specific myeloid malignancies is generally limited and inconsistent. Future epidemiological studies should report findings for the specific myeloid malignancies, as combining them post hoc – where appropriate – always remains possible, whereas disaggregation may not. Furthermore, combining results across possibly discrete diseases reduces the chances of identifying important malignancy-specific causal associations.

**Supplementary Information:**

The online version contains supplementary material available at 10.1186/s12885-021-07908-3.

## Introduction

Hematopoietic and lymphoid malignancies (also known as lymphohematopoietic malignancies, or LHM) arise from stem and progenitor cells derived from hematopoietic stem cells. These diseases, though, represent several heterogeneous groups of neoplasms that are biologically, etiologically or clinically distinct [1]. LHM are classified based on the progenitor cells from which they arise, the vast majority being of lymphoid (i.e., derived from the lymph and lymphatic system) or myeloid (deriving from the bone marrow) origin, although much rarer malignancies may arise from dendritic or histiocytic cells.

Lymphoid malignancies generally are associated with lymphoid progenitor cells that mature into cells of the immune system, including B lymphocytes [B-cells], T lymphocytes [T-cells], and Natural Killer [NK] cells), but are categorized by the stage of differentiation of the tumor cells rather than the cell in which the initial transforming event occurred [2]. Lymphoid malignancies include various lymphomas, as well as acute lymphoblastic leukemia (ALL) and chronic lymphocytic leukemia (CLL). Myeloid malignancies arise from myeloid progenitor cells and include all granulocytic (e.g., erythrocytes, or red blood cells) and mast cell lineages [3]. Myeloid malignancies include myelodysplastic syndrome (MDS), acute myeloid leukemia (AML, which has replaced the term acute nonlymphocytic leukemkia, ANLL), myeloproliferative neoplasms (MPN), chronic myeloid (or “myelogenous”) leukemia (CML) – and myelodysplastic/myeloproliferative neoplasms (MDS/MPN) [2]. Multiple myeloma is a malignant disorder involving plasma cells which originate from B-cells. Most of these sub-groups of LHM contain multiple entities with diverse etiologies and possible underlying risk factors.

The 2008 revision of the World Health Organization (WHO) classification of LHMs led to changes in the classification of leukemias and especially myeloid leukemias for epidemiological research based on improved understanding of the lineage of the cells, as well as the molecular genetics and pathologic characteristics of the different malignancies. The WHO classification was further updated in 2016 for lymphoid [4] and for myeloid malignancies [5].

The primary objective of this paper is to evaluate the published epidemiological evidence on the myeloid malignancies for chemical agents classified by the International Agency for Research on Cancer (IARC) as Group 1 carcinogens (that is, “carcinogenic to humans,” commonly referred to as “known human carcinogens”) and for which the epidemiological evidence of a causal association was considered sufficient. The epidemiological and toxicological evidence for associations with exposure to certain chemicals (e.g., benzene) appears to be stronger for specific myeloid malignancies – especially AML and MDS – than for leukemias as a group or lymphoid malignancies, which are generally more closely related to infections and immunological functions [6].

### PART I: overview of the myeloid malignancies

Since 2001, the WHO has included genetic information relevant to the diagnosis and classification of LHMs, and the 2008 WHO classification of myeloid neoplasms built on the 2001 classification. The underlying pathology in myeloid malignancies is based on clonal proliferations arising in hematopoietic stem or progenitor cells, and specific diseases are often associated with genetic or epigenetic changes in genes involved in regulation of cell growth. The 2016 update to the 4th Edition of the WHO Classification of Tumors of the Hematopoietic and Lymphoid Tissues additionally incorporated clinical features, morphology, immuno-phenotyping, cytogenetics, and molecular genetics to classify both acute and chronic myeloid leukemias into subtypes and discrete disease entities of clinical significance [7]. A brief review of the current pathology and classification of the myeloid malignancies illustrates several ways in which specific myeloid malignancies differ and a basis for epidemiologically examining them separately (Part II).

#### Myelodysplastic syndromes (MDS)

MDS refers to a heterogeneous collection of clonal disorders of pluripotent hematopoietic progenitor cells (HPC) that demonstrate lower than normal blood cell counts (cytopenias), an increased percentage of blasts in bone marrow, and dysplasia in erythroid cells, granulocytes, or megakaryocytes [5]. MDS generally has an insidious onset, often diagnosed due to vague symptoms arising as a manifestation of cytopenias, and a variable prognosis, depending upon the molecular genetic profile of the subtype and individual response to therapy. Approximately 20–30% of MDS patients over the age of 65 go on to develop AML, suggesting that at least some proportion of these cases may represent the same underlying disease processes or share causal factors [8]. Some acquired mutations seen in the development of MDS include those in genes involved in RNA splicing (*SRSF2*), DNA methylation (*DNMT3a, TET2, IDH 1/2*), chromatin modification (*ASXL1*) or the cohesion complex (*STAG2*) [9].

MDS is more prevalent in older adults, with the majority of cases diagnosed in individuals over the age of 60 [10]. Rates of MDS appear to be increasing, which may be due to improvements over time in diagnostic specificity combined with clearer diagnostic criteria for MDS [11].

#### Acute myeloid leukemia (AML)

The classification of AML includes 20 definitive and 2 provisional subtypes [5]. AML generally has a rapid onset, often diagnosed due to the development of infections, bleeding, or fatigue that result from pancytopenia, and a variable prognosis, depending upon the molecular genetic profile of the subtype and individual response to therapy.

AML is a genetically diverse disease, with 40–55% of patients having chromosome abnormalities that can be identified using conventional analysis techniques [12–14]. The most common genetic change is the loss of genetic material in chromosome 5 or chromosome 7 [13]. Others include deletions in parts of chromosomes (e.g., the long arms of chromosomes 5, 7, and 9), insertion of genetic material, inversions of genetic material (e.g., involving chromosome 16), duplications, and translocations (e.g., t[8;21], t[15;17], and 11q23 translocation]) [13]. Approximately 40–50% of AML patients have a normal karyotype and harbour mutations within specific genes including *IDH1*, *IDH2*, *FLT3*, and *NPM1*.

Some AMLs develop secondary to MDS, and these occur in patients with acquired mutations in genes encoding for myeloid transcription factors (*RUNX1*, *CEBPA*) or signal transduction proteins (*FLT3*) [9]. However, de novo AMLs are also diagnosed in patients with mutations in *RUNX1*, *CEBPA*, *FLT3* or *MLL*, but these patients do not have mutations in the genes associated with prior MDS (described above) [9]. Estey (2018) estimated that one-third of patients clinically diagnosed with de novo AML will exhibit genetic mutations specific for secondary AML [9].

AML is more common in the elderly, with more than 58% of cases diagnosed among those 65 years of age or older [10].

#### Myeloproliferative neoplasms (MPN)

MPNs (previously known as myeloproliferative disorders, or MPD) are a group of clonal hematopoeitic neoplasms, including polycythemia vera (PV), essential thrombocythemia (ET), and myelofibrosis (MF). These conditions are associated with the proliferation of one or more of the myeloid lineages (i.e., increased blood cell counts), without dysplasia. CML shares several features with these disorders, e.g., dysregulated production of a particular lineage of mature myeloid cells, a tendency to progress to acute leukemia, and abnormalities in thrombosis and hemostasis. Many diagnoses of MPNs occur in patients that have acquired mutations in the Janus kinase 2 (*JAK2)* gene, seen in 95% of patients diagnosed with PV and over 50% of patients diagnosed with MF and ET) [15]. Other mutations seen in patients with MPN include calreticulin (*CALR*), myeloproliferative leukemia virus oncogene (*MPL*) [16].

SEER data are limited for MPN, however, ET represented 45.5% of the cases and PV accounted for 41.5% of the cases. The incidence rate was slightly higher in males compared to females, 3.3 vs. 3.0 per 100,000, respectively. Incidence rates increased with age from 0.5 per 100,000 for under age 40 to 18.6 per 100,000 for ages 80 and over [10].

#### Chronic myeloid leukemia (CML)

In CML, the proliferating cells are mature cells of the myeloid lineage, which have differentiated into functional formed elements of the blood. The development of CML involves an acquired cytogenetic abnormality in the pluripotent hematopoietic stem cells (HSCs) or myeloid progenitor cells located in the bone marrow. Ninety-five percent of CML cases involve the reciprocal translocation of genetic material between chromosome 22 and chromosome 9 [t(9;22)(q34;q11)]. This translocation results in an abnormally shortened version of chromosome 22, known as the “Philadelphia (Ph) chromosome” [17, 18].

In the United States, the median age at diagnosis of CML was 65 years, while the median age at death was 77 years. The incidence rate among males, for all races and ethnicities and all age-groups, was 2.4 per 100,000 population, while among females the rate was 1.4 per 100,000. Incidence among white males was 2.5 per 100,000 while the incidence was 2.2 per 100,000 among black males. Incidence among those under 65 years of age was 1.1 per 100,000 population, but nearly seven times higher (i.e., 7.6 per 100,000) among those 65 and over. Incidence among the population aged 65 and over was highest among white males (11.1 per 100,000), followed by black males (8.2 per 100,000), white females (5.7 per 100,000) and black females (4.9 per 100,000) [10].

#### Myelodysplastic syndrome/Myeloproliferative Neoplams (MDS/MPN)

The 2016 Classification of LHM includes a category for MDS/MPN. These neoplasms are characterized by both dysplastic and proliferative features. Examples include chronic myelomonocytic leukemia (CMML), atypical chronic myeloid leukemia (aCML) and juvenile myelomonocytic leukemia (JMML) [7]. However, these specific myeloid neoplasms are very rare and infrequently considered in epidemiological studies; therefore, they are not discussed further.

### PART II: epidemiological evaluation of four environmental agents and specific myeloid malignancies

#### Methods

We reviewed the list of carcinogenicity classifications by cancer site published on the IARC Monographs website [19, 20]. The IARC has identified 28 agents as having sufficient evidence of carcinogenicity in humans for neoplasms the IARC grouped as “leukemia and/or lymphoma”. We assessed the human evidence summaries in the “Evaluation” sections of the relevant IARC monographs for each agent.

We excluded from our review 10 pharmaceutical agents (azathioprine, busulfan, chlorambucil, cyclophosphamide, etoposide with cisplatin and bleomycin, melphalan, MOPP [vincristine-prednisone-nitrogen mustard-procarbazine], semustine [methyl-CCNU], thiotepa, and treosulfan) because most of these are chemotherapy agents in which exposure is voluntary and the expected benefit likely offsets the possible leukemogenic effect. We also eliminated radioactive (e.g., X- and gamma radiation, fission-products radionuclides [including strontium-90], thorium-232 and its decay products) and microbiological (Epstein Barr virus, helicobacter pylori, hepatitis C virus, Human immunodeficiency virus type 1, Human T-cell lymphotropic virus type 1, Kaposi sarcoma herpes virus) agents. We excluded two pesticides - pentachlorophenol and lindane - because IARC identified the human evidence as sufficient for causing NHL (lymphomas). We also excluded IARC’s evaluation “occupational exposures in the rubber-manufacturing industry” because workers in the industry are exposed to multiple chemicals and it cannot be determined which specific agents may be causally related to leukemia.

After these exclusions, four leukemogenic chemical agents remained: 1,3-butadiene, formaldehyde, benzene and tobacco smoking. For each of these, we conducted a focused systematic review of the literature using searches of the relevant IARC Monographs and key word searches of PubMed to identify epidemiological studies that reported results separately for specific subtypes of myeloid malignancies. Keywords included “benzene”, “1,3-butadiene,” “formaldehyde,” “cigarette,” “smoking,” “leukemia,” “myeloid,” “AML,” “CML,” “MDS,” and “MDN.” Where results of independent studies of acceptable quality were available, we conducted meta-analyses using random-effects models [21]. For each study, the following characteristics were extracted consistent with PRISMA guidelines [22]: study design, study population, geographic location, study period, exposure categories, number of deaths observed or number of cases in exposed and unexposed groups, relative risk measures (SMRs, HRs, RRs, and ORs) 95% confidence intervals (CI) and covariates adjusted for in models. Using meta-analysis, summary relative risk estimates were calculated by specific categories of myeloid malignancy including AML, CML and MDS. Cohort studies and case-control studies were analysed separately as well as overall and where possible for the highest exposure categories. When multiple results were published on the same study population, we preferentially selected for meta-analysis those based on incidence data, those representing the most complete results, or results reported for higher exposure categories. Publication bias was assessed using a visual inspection of the funnel plots as well as Egger’s test (see supplemental file). Heterogeneity was evaluated using the I^2^ statistic, which provides a measure for quantifying inconsistency of effects across studies. All meta-analyses were conducted using R version 3.6.1 (2019-07-05).

## Results

### 1,3-butadiene (butadiene)

The IARC last reviewed the carcinogenicity of butadiene in 2009 [23]. The epidemiological evidence for exposure to butadiene and risk of leukemia is based primarily on studies conducted among workers in the butadiene monomer industry and workers in the styrene–butadiene rubber (SBR) manufacturing industry. However, results on specific types of leukemia are available only from studies conducted in the SBR manufacturing industry.

A study of approximately 17,000 workers from eight SBR facilities across the United States and Canada reported an increased risk of leukemia among 16,610 workers (12,412 exposed to butadiene), based on 58 leukemia deaths [24]. Because standardized mortality ratio analyses were not conducted, it is not clear whether excess mortality from leukemias occurred. A positive dose-response was reported between cumulative exposure to butadiene and risk of leukemia. Despite the individual exposure estimates and the relatively large number of leukemia deaths, results by leukemia subtype were not reported.

The mortality follow-up was extended through 1998 for 15,649 men employed since 1943, 75% of whom were exposed to butadiene [25]. A total of 71 deaths from leukemia was observed (SMR 1.16, 95% CI, 0.91–1.47). No consistent patterns were observed by categories of years since hire or by years worked. The excess leukemia mortality was concentrated among men hired in the 1950s (31 deaths; SMR, 1.50; 95% CI, 1.01–2.11). In the analysis by leukemia subtype the SMR was 1.02 (95% CI 0.56–1.71, 14 deaths) for AML and 1.67 (95% CI 0.83–2.99, 11 deaths) for CML. Mortality from AML was elevated in maintenance laborers and from CML in laboratory workers; however these were based on only five and three deaths, respectively.

Time-dependent exposure-response relationships between several butadiene exposure indices and leukemia (81 decedents) as well as all myeloid neoplasms (56 decedents from myeloid and monocytic leukemia, myelofibrosis, myelodysplasia, myeloproliferative disorders and polycythemia vera) were evaluated [26]. The butadiene exposure indices included cumulative exposure in ppm–years, total number of exposures to peaks (> 100 ppm) and average intensities of exposure in parts per million. All three exposure indices were associated positively with the risk for leukemia whereas the myeloid neoplasms were more clearly associated with peak exposures. This highlights the potential additional role choice of exposure metric may play in evaluating risk [27].

Only two studies evaluated the risk of myeloid malignancies [25, 26], based on the same study cohort. Risks of AML were not increased, and risk of CML was increased but not statistically significantly. For myeloid leukemias (including CML), a relationship was reported for peak exposure, but not cumulative exposure.

### Formaldehyde

The IARC last reviewed the carcinogenicity of formaldehyde in 2009 (23). The epidemiological literature on exposure to formaldehyde and risk of leukemia published since the IARC meeting was reviewed in detail [28]. Twenty studies that reported results for leukemia overall were included, three of which also reported results for myeloid leukemia. Since then, some of the occupational epidemiological studies have been updated or re-analyzed, and new studies have been published that examine myeloid leukemias in relation to formaldehyde exposure (Table 1).

**Table 1:** Select characteristics of included formaldehyde studies

Reference	Study design	Population or Cases / controls	Study setting	Years of follow-up	Adjusted for smoking
Meyers 2013 [29]	Occupational cohort	11,098 garment manufacturing workers	US: Georgia and Pennsylvania	1960–2008	No
Coggon 2014 [30]	Occupational cohort	14,008 chemical factory workers	UK: England and Wales	1941–2012	No
Checkoway 2015 [31]	Occupational cohort	25,619 workers at formaldehyde using or producing plants	US: Re-analysis of Beane Freeman 2009	1943–2004	No
Beane Freeman 2009 [32]	Occupational cohort	25,619 workers at formaldehyde using or producing plants	US: 10 industrial plants	1943–2004	No
Saberi Hosjineh 2013 [33]	Population-based cohort	241,465 adults (European Prospective Investigation into Cancer and Nutrition cohort)	10 European countries: Denmark, France, Greece, Germany, Italy, The Netherlands, Norway, Spain, Sweden, and the UK	1992–2010	Yes
Talibov 2014 [34]	Population-based case-control	14,982 AML cases 74,505 controls	4 Nordic countries: Finland, Norway, Sweden, and Iceland	1960–2005^a^	No

^a^ Study period varies by country

Peak exposure in a cohort of workers employed in six plants producing formaldehyde in the United States was re-defined and analysed with respect to specific leukemia types. Absolute peak exposure, duration of time worked at the highest peak or time since highest peak exposure generated no clear associations with myeloid leukemia or AML. Cumulative exposure also was unrelated to risk of leukemia, myeloid leukemia, AML, or CML. The authors concluded, “Findings from this re-analysis do not support the hypothesis that formaldehyde is a cause of AML” [31]. The use of peak exposure in this and other epidemiological studies presents specific challenges that have been explored separately [27].

The other occupational cohort study of formaldehyde producers also reported no clear associations between different metrics of formaldehyde exposure and myeloid leukemia [30]. In a study of garment workers in the United States [29, 35], moderately elevated relative risks for myeloid leukemia were associated with duration of employment, a surrogate for cumulative exposure, and duration of follow-up, a surrogate for latency. A large cancer registry study in the Nordic countries “did not provide clear evidence for an association between occupational solvent exposure and AML” [34].

There were no deaths from myeloid leukemias among a cohort of laminated plastic workers from Italy [36]. A European community-based cohort study [33] found no increased risks of AML or CML among study subjects with low-level occupational exposure to formaldehyde (no study subjects were reported to have high occupational exposure to formaldehyde).

SMR results for myeloid leukemia, AML and CML, including those for the highest categories of exposure from the most recent updates of the industrial cohorts, are summarized in Table 2. Overall, the updated cohort study analyses demonstrate no clear or consistent excess risk of myeloid leukemia or AML or CML. None of the formaldehyde studies evaluated MDS or MPN. Table 3 presents meta-analysis results by myeloid malignancy, specifically ML, AML and CML. No statistically significant increased meta-relative risk estimates were seen. Based on the I^2^ test, heterogeneity was low, and based on Egger’s test, publication bias appears unlikely.

**Table 2:** Formaldehyde exposure and risk of specific types of myeloid malignancy by exposure category

Reference	Exposure Category	Myeloid leukemia			AML			CML		
		No. of cases	Point estimate	95% CI	No. of cases	Point estimate	95% CI	No. of cases	Point estimate	95% CI
Overall Results in Most Informative Cohorts										
Meyers 2013 [29]	Exposed	21	1.28	0.79–1.96	14	1.22	0.67–2.05	5	1.35	0.44–3.15
Coggon 2014 [30]	Exposed	36	1.20	0.84–1.66						
Checkoway 2015 [31]	Exposed	44	0.86	0.64–1.16	30	0.80	0.56–1.14	13	0.97	0.56–1.67
Results of Category at Highest Exposure in Studies										
Beane Freeman 2009 [32]	Peak exposure > 4 ppm	19	1.78	0.87–3.64						
Checkoway 2015 [31]	Peak exposure > 4 ppm	10	1.80	0.85–3.79	6	1.43	0.56–3.63	4	3.07	0.83–11.40
	Cumulative exposure > 2.5 ppm-yrs	14	0.94	0.47–1.86	10	0.96	0.43–2.16	4	0.92	0.25–3.36
Coggon 2014 [30]	High exposure, > one year	50	0.96	0.24–3.82						
Saberi Hosjineh 2013 [33]	≥Low exposure				N/A	1.01	0.65–1.57	N/A	0.92	0.46–1.84
Meyers 2013 [29]	Duration of exposure 10 + yrs.	10	1.84	0.88–3.38	7	1.81	0.73–3.73			
Talibov 2014 [34]	Cumulative exposure > 1.6 ppm-yrs				424	1.17	0.91–1.51

**Table 3:** Meta-analysis of formaldehyde exposure and risk of specific types of myeloid leukemia in the most informative cohorts

Characteristics	No. of estimates	Meta-RR estimate (95%CI)	*I*^*2*^ test (%)	p-value	Egger’s test
**ML**					
High exposure	3	1.28 (0.81–2.02)	0.00	0.2874	0.7851
Any exposure	3	1.05 (0.85–1.30)	8.85	0.6622	0.4056
**AML**					
High exposure	4	1.15 (0.94–1.41)	0.00	0.1808	0.8301
Any exposure	2	0.90 (0.67–1.22)	0.00	0.5063	NA
**CML**					
High exposure	2	0.92 (0.50–1.70)	0.00	0.7893	NA
Any exposure	2	1.05 (0.65–1.69)	0.00	0.8458	NA

### Benzene

The IARC last reviewed the carcinogenicity of benzene in 2018 [6]. Risk estimates for one or more myeloid malignancies were reported in 31 independent studies. Characteristics of the design of these studies are summarized in Table 4, and results are summarized in Table 5.

**Table 4:** Select characteristics of included benzene studies

Reference	Study design	Population or Cases / controls	Study setting	Years of follow-up	Adjusted for smoking
Adegoke 2003 [37]	Population-based case-control	236 AML cases; 79 CML cases 502 controls	China: Shanghai	1987–1989	No
Albin 2003 [38]	Population-based case-control	330 MDS cases 337 controls	Southern Sweden	1976–1993	No^a^
Blair 2001 [39]	Population-based case-control	132 AML cases; 46 CML cases; 58 MDS cases 1087 controls	USA: Iowa and Minnesota	1976–1993	Yes
Bjork 2001a [40]	Population-based case-control	226 CML cases 251 controls	Southern Sweden	1980–1984	No*
Bonzini 2019 [41]	Occupational cohort	5112 oil refinery workers	Italy	1976–1993	No
Collins 2015 [42]	Occupational cohort	2266 chemical workers	USA: Michigan	1944–1977	No
Copley 2017 [43]	Hospital-based case control	604 MDS cases 1193 controls	China: Shanghai	2003–2007	No^a^
Costantini 2008 [44]	Population-based case-control	142 AML cases 893 controls	Italy	1991–1993	No^a^
Divine 1999 [45]	Occupational cohort	28,480 oil refinery and petrochemical workers	USA: Texas	1947–1993	No
Divine 2000 [46]	Occupational cohort	24,124 oil production and pipeline workers	USA	1946–1994	No
Guenel 2002 [47]	Nested case-control	26 AML cases 103 controls	France	1978–1989	No
Huebner 2009 [48]	Occupational cohort	127,266 petroleum workers	USA: Louisiana and Texas	1979–2000	No
Ireland 1997 [49]	Occupational cohort	4172 chemical workers	USA: Illinois	1940–1991	No
Kirkeleit 2008 [50]	Occupational case-control	27,919 upstream petroleum workers	Norway	1981–2003	No
Linet 2015 [51]	Occupational cohort	73,789 benzene-exposed and 34,405 unexposed workers	China	1972–1999	No
McCraw 1985 [52]	Occupational cohort	3976 oil refinery workers	USA: Illinois	1973–1982	No
Poynter 2017 [53]	Population-based case-control	420 AML cases; 265 MDS cases 1388 controls	USA: Minnesota	2010–2014	Yes
Rhomberg 2016 [54]	Occupational cohort	1696 Pliofilm workers	USA: Ohio	1940–1996	No
Rushton 2014 [55]	Nested case-control	60 AML cases 241 controls	Canada, UK, and Australia	1950–2006^c^	No
Saberi Hosnijeh 2013 [33]	Population-based cohort	241,465 adults (European Prospective Investigation into Cancer and Nutrition cohort)	10 European countries: Denmark, France, Greece, Germany, Italy, The Netherlands, Norway, Spain, Sweden, and the UK	1992–2010	Yes
Sathiakumar 1995 [56]	Occupational case-control	10 AML cases; 5 CML cases; 42 controls	USA	1976–1990	No
Satin 1996 [57]	Occupational cohort	17,844 Port Arthur oil refinery workers	USA: Texas	1937–1987	No
Schnatter 2012 [58]	Occupational case-control	29 MDS cases; 60 AML cases; 30 MPD cases; 28 CML cases 616 controls	Canada, UK, and Australia	1950–2006^c^	No^a^
Stenehjem 2015 [59]	Occupational cohort	4 MDS cases; 10 AML cases; 3 CML cases ^b^ 1661 controls	Norway	1999–2011	Yes
Strom 2005 [60]	Hospital-based case-control	354 MDS cases 452 controls	USA: Texas	1999–2003	Yes
Talibov 2014 [34]	Population-based case-control	14,982 AML cases 74,505 controls	4 Nordic countries: Finland, Norway, Sweden, and Iceland	1960–2005^c^	No
Teras 2019 [61]	Population-based cohort	115,996 adults (American Cancer Prevention Study-II Nutrition cohort)	USA	1997–2013	Yes
Wong 1993 [62]	Occupational cohort	18,135 gasoline distribution workers	USA	1946–1986	No
Wong 2001a [63]	Occupational cohort	7543 petroleum refinery workers	USA: Texas	1945–1996	No
Wong 2001b [64]	Occupational cohort	3328 petroleum refinery workers	USA: California	1959–1997	No
Wong 2010 [65]	Hospital-based case-control	722 AML cases 1444 controls	China: Shanghai	2003–2007	No

^a^ Statistical model was not adjusted for smoking status, however smoking status was investigated and reported independently

^b^ MDS and CML were not analyzed

^c^ Study period varies by country

**Table 5:** Results of studies on benzene exposure and specific type of myeloid malignancy*

Reference	Exposure category	AML		CML		MDS		MPN		Myeloid leukemia	
		Point estimate	95% CI	Point estimate	95% CI	Point estimate	95% CI	Point estimate	95% CI	Point estimate	95% CI
Adegoke 2003 [37]	> 15 yrs	2.9	1.2–7.0	5	1.8–13.9						
Albin 2003 [38]	High exp					0.95	0.54–1.7				
Blair 2001 [39]	High exp.	1.1	0.3–3.9	0	–	2.6	0.7–9.7				
Bjork 2001a [40]	Exposed			1.2	0.66–2.3						
Bonzini 2019 [41]	20+ years	2.05	0.66–6.36	1.34	0.17–8.80					1.98	0.82–4.75
Collins 2015 [42]	25+ ppm-yrs	1.39	0.17–5.03			25.1	0.63–139.6			1.93	0.53–4.94
Copley 2017 [43]	> 12 ppm					3.47	1.65–7.28				
Costantini 2008 [44]	M/H exp	0.9	0.4–2.3								
Divine 1999 [45]	Exposed	1.29	0.79–1.99	1.05	0.54–1.83						
Divine 2000 [46]	Exposed	1.92	1.10–3.13	0.94	0.34–2.05						
Guenel 2002 [47]	> 5.5 unit-yrs	2.4	0.7–8.5	1.2	0.1–11.4						
Huebner 2009 [48]	Exposed	1.07	0.84–1.36	1.01	0.67–1.47	1.64	0.78–3.01				
Ireland 1997† [49]	≥ 72 ppm-mo	4.5	0.1–25.3								
Kirkeleit 2008 [50]	Exposed	2.89	1.25–6.67	1.44	0.19–10.7						
Linet 2015 [51]	Exposed	2.1	0.9–5.2	2.5	0.8–10.7	∞	1.9-∞			2.2	1.1–4.6
McCraw 1985 [52]	Exposed	3.94	1.72–7.88	1.21	0.02–6.96						
Poynter 2017 [53]	≥ 5 years	1.77	1.19–2.63			2.10	1.35–3.28				
Rhomberg 2016 [54]	≥ 80.11 ppm-yrs (0 lag)	10.11	3.71–22.01								
	≥ 74.55 ppm-yrs (10 yr lag)	10.76	3.95–23.43								
	≥ 73.53 ppm-yrs (20 yr lag)	6.37	1.31–18.61								
Rushton 2014 [55]	> 2.93 ppm-yrs	1.39	0.68–2.85								
	> 0.259 ppm	1.90	0.86–4.18								
	> 28 yrs	1.70	0.75–3.87								
	> 0.413 max ppm	1.65	0.75–3.63								
Saberi Hosnijeh 2013 [33]	Exposed	1.52	0.78–1.81	1.97	0.75–5.19					1.60	0.95–2.69
Sathiakumar 1995 [56]	Exposed	2.8	1.1–7.3	1.6	0.43–5.9					2.1	1.0–4.3
Satin 1996 [57]	Exposed	0.63	0.30–1.15	0.84	0.31–1.84						
Schnatter 2012 [58]	> 2.93 ppm-yrs			2.20	0.63–7.68	4.33	1.31–14.3	1.79	0.68–4.74		
	> 0.259 ppm			0.91	0.25–3.28	3.12	0.90–10.8	1.17	0.39–3.52		
	> 28 yrs			1.42	0.35–5.67	1.74	0.61–4.99	0.82	0.25–2.73		
	> 0.413 max ppm			0.59	0.15–2.37	2.81	0.79–10.1	0.85	0.28–2.61		
Stenehjem 2015 [59]	≥0.124 ppm-yrs	4.85	0.88–27.0							2.24δ§	0.65–7.71
	≥13 yrs	0.97	0.08–11.0							1.47§	0.37–5.88
	> 0.013 ppm	3.47	0.63–19.0							1.66§	0.48–5.74
Strom 2005 [60]	High exp					2.05	1.20–3.51				
Talibov 2014 [34]	> 13.6 ppm-yrs	0.80	0.56–1.15								
Teras 2019 [61]	≥1.49 μg/m3	0.98	0.68–1.42			1.28	0.86–1.92			0.89	0.67–1.19
Wong 1993 [62]	Exposed (marine)	0.74	0.24–1.73	1.33	0.36–3.4						
	Exposed (land)	1.51	0.80–2.57	0.26	0.01–1.43						
Wong 2001a [63]	Exposed	1.47	0.76–2.57	1.31	0.43–3.07						
Wong 2001b [64]	Exposed	0.45	0.01–2.53	1.96	0.24–7.07						
Wong 2010 [65]	Exposed	1.43	1.05–1.93								

* Only studies reporting results on specific myeloid malignancy types are reported. †Study reports acute-nonlymphocytic leukemia (ANLL). ‡RR estimate is for MDS/AML combined. §Myeloid malignancies including MDS. Four cases of MDS were reported, and MDS was not analyzed separately

Abbreviations: *NA* Not available; *M/H* Medium/high; *exp*. Exposure; *mo* Months; *ppm* Parts per million; *yrs*. Years;

A cohort exposed to benzene in a variety of manufacturing and user industries, including paints and painting, printing, footwear, paints, chemicals in 12 cities in China was followed for mortality. In the most recent update of this cohort, 73 leukemia deaths were observed, including 60 among benzene-exposed workers [51]. Similar risks were reported for AML and CML, while the risk of MDS was inestimable due to zero cases of MDS among the unexposed group (Table 5). A case-cohort analysis of combined AML/MDS (44 cases) and CML (18 cases) from the 12-city China cohort examined the timing of exposure [66]. The investigators found that high cumulative exposure or high intensity exposure experienced 2 to 10 years before diagnosis increased the risk of MDS/AML among workers who were first exposed under 30 years of age, but not for workers first exposed 30 years of age or older [66].

Pooled results for AML, CML, MDS and MPN were reported using data from three separate nested case-control studies of petroleum workers from Canada, the UK, and Australia. No significantly elevated risks of AML by cumulative exposure, average exposure intensity, maximum exposure intensity, duration of employment, and peak exposure were reported [55]. Increased relative risk of MDS for cumulative exposure greater than 2.93 ppm-years and peak exposure less than 3 ppm were reported, but not for CML or MPN [58]. The most recent follow-up of incidence in the UK petroleum distribution and oil refinery workers reported deficits of MDS [67]. Similarly, the most recent mortality follow-up of the Canadian Petroleum Workers cohort reported no excess of MDS [68].

An increased mortality risk of MDS associated with 25 ppm-years or more of benzene exposure was reported, however, this finding was based on only one death [42]. A much larger registry-based study of occupational exposure to benzene and incidence of AML indicated no increased risk [34]. Age-stratified analyses indicated a possible increased risk of AML in workers under age 50 and in the highest benzene exposure group [34].

Since studies did not report results for the same subtypes of leukemia, it is problematic to combine all results using meta-analysis. We, therefore, conducted meta-analyses of results reported for myeloid leukemias combined or specifically for AML, MDS and CML (Table 6). The meta-analysis of results for AML was based on 27 estimates from 26 publications and generated a summary RR of 1.30 (95% CI 1.09–1.55; I^2^ = 48.91%) with similar increases, but some variation in the summary RR across exposure categories (i.e., high, low, any exposure). The results for low and any exposure to benzene were not statistically significantly elevated. Egger’s test was significant for the overall result and for cohort studies, but not for the other meta-analyses (Table 6). Visual inspection of funnel plots indicated possible publication bias favoring negative results (see supplemental file).

**Table 6:** Meta-analyses of studies of specific myeloid maligancy type for benzene.*

Characteristics	No. of estimates	Meta-RR estimate (95%CI)	I^2^ test (%)	p-value	Egger’s test
**ML**					
Overall	7	1.56 (1.10–2.20)	45.06	0.0114	0.0063**
High exposure	4	1.28 (0.87–1.88)	40.98	0.2051	0.0984
Low exposure	1	2.24 (0.65–7.71)	NA	0.2012	NA
Any exposure	2	2.15 (1.29–3.58)	0.00	0.0033	NA
Study type					
Case-control studies	0				NA
Cohort studies	7	1.56 (1.10–2.20)	45.06	0.0114	0.0063**
**AML**					
Overall	27	1.30 (1.09–1.55)	48.91	0.0037	0.0155**
High exposure	8	1.65 (1.13–2.41)	46.97	0.0100	0.1176
Low exposure	5	1.54 (0.89–2.66)	58.71	0.1248	0.2679
Any exposure	14	1.14 (0.93–1.39)	36.29	0.2057	0.3933
Study type					
Case-control studies	9	1.34 (1.03–1.75)	40.12	0.0281	0.5929
Cohort studies	18	1.29 (1.02–1.63)	51.98	0.0364	0.0119**
**CML**					
Overall	18	1.25 (1.00–1.55)	0.00	0.0456	0.2347
High exposure	3	2.79 (1.44–5.40)	0.00	0.0024	0.7110
Low exposure	2	1.93 (0.64–5.82)	0.00	0.2447	NA
Any exposure	13	1.11 (0.87–1.40)	0.00	0.4019	0.4132
Study type					
Case-control studies	5	1.93 (1.05–3.56)	25.76	0.0353	0.6999
Cohort studies	13	1.13 (0.89–1.45)	0.00	0.3215	0.3540
**MDS**					
Overall	9	1.87 (1.39–2.52)	40.73	< 0.0001	0.0560
High exposure	6	1.80 (1.18–2.75)	51.97	0.0065	0.1173
Low exposure	2	2.29 (1.51–3.48)	0.00	< 0.0001	NA
Any exposure	1	1.64 (0.83–3.22)	N/A	0.1510	NA
Study type					
Case-control studies	5	1.85 (1.28–2.67)	33.43	0.0012	0.5538
Cohort studies	4	1.94 (1.19–3.18)	43.78	0.0081	0.1088

*MPN not included as only one published point estimate was identified.**statistically significant (*p*. < 0.05)

For CML, the meta-analysis of overall results was based on 18 estimates from 17 studies resulting in a summary RR of 1.25 (95% CI 1.00–1.55; I^2^ = 0%) with large variation in the summary RR across exposure categories. Publication bias appears unlikely. The meta-RR for myeloid leukemias combined, based on seven studies, was 1.56 (95% CI 1.10–2.20; I^2^ = 45.06%) with wide variability by exposure category (high, low, any exposure). Evidence of publication bias was present for the overall and cohort meta-analyses, but small numbers of studies hindered results for the exposure categories (Table 6). Visual inspection of funnel plots indicated that publication bias favored positive results (see supplemental file).

The meta-analysis for MDS was based on nine studies and generated a summary RR of 1.87 (95% CI 1.39–2.52; I^2^ = 40.73%) with similar risks for the low exposure category (m-RR = 2.29, 95% CI 1.51–3.48, I^2^ = 0%) and the high exposure category (m-RR = 1.80, 95% CI 1.18–2.75, I^2^ = 51.97%). Publication bias appears unlikely. The meta-analyses for the overall category for each outcome were also calculated by study type (Table 6). The meta-analyses for CML by study type revealed a large difference between the case-control (m-RR = 1.93; 95% CI 1.05–3.56, I^2^ = 25.76%) and cohort studies (m-RR = 1.13; 95% CI 0.89–1.45, I^2^ = 0%), possibly reflecting reporting bias, as many of the case-control studies were population-based and dependent on self-reported exposure. This difference by study design was not observed for AML, MDS or the category of all myeloid leukemias combined.

The interpretation of results on risk of specific leukemia types from exposure to benzene is complicated by the heterogeneity in exposure circumstances. However, the evidence indicates a similar association between occupational exposure to benzene and specific myeloid neoplasms, but the association appears strongest for MDS, especially among more recent studies. This raises the question of whether earlier studies identifying associations between generally very high benzene exposure and AML might have reflected the occurrence of secondary AML following unrecognized (or misdiagnosed) primary cases of MDS. It is noteworthy that a very large record linkage study from the Nordic countries reported no association between benzene exposure and AML incidence [34].

### Tobacco smoking

The IARC last evaluated the carcinogenicity of tobacco smoking in 2009 [69]. From the IARC review and the PubMed search, we identified 42 studies on tobacco smoking and risk of myeloid malignancies, 27 of which reported results for current or ever smokers (current and former smokers combined) as summarized in Table 7. The remaining studies reported results for different groups of smokers, defined according to dose (cigarettes per day), duration (years of smoking) or cumulative consumption (pack-years). Whenever possible, we selected results by duration of smoking, since this is the exposure metric most strongly associated with lung cancer risk (Table 8).

**Table 7:** Select characteristics of tobacco smoking studies

Reference	Study design	Population (cohort) Cases/controls	Study setting	Study period
Averginou 2017 [70]	Hospital-based case-control	126 MDS cases 102 controls	Greece	2009–2013
Batty 2008 [71]	Occupational cohort	17,322 government workers	London	1967–2005
Bjork 2000 [72]	Population-based case-control	330 MDS cases 337 controls	Southern Sweden	1976–1993
Bjork 2001b [73]	Population-based case-control	284 AML cases 332 controls	Southern Sweden	1976–1993
Bjork 2009 [74]	Population- and hospital-based case-control	79 MDS cases; 104 AML cases 278 controls	Southern Sweden	2001–2004
Brown 1992 [75]	Population-based case-control	178 AML cases; 65 CML cases 1742 controls	Iowa and Minnesota	1981–1984
Brownson 1991 [76]	Registry-based case-control	M: 189 AML cases; 88 CML cases 1899 controls F: 178 AML cases; 65 CML cases 1742 controls	Missouri	1984–1990
Dalamaga 2002 [77]	Hospital-based case-control	84 MDS cases 84 controls	Greece	1995–2000
Fernberg 2007 [78]	Occupational cohort	336,381 construction workers	Sweden	1969–2004
Ido 1996 [79]	Hospital-based case-control	116 MDS cases 116 controls	Japan	Sep-Oct 1992 and Aug-Oct 1993
Kabat 1988 [80]	Hospital-based case-control	249 ANLL cases; 78 CML cases 9342 non-cancer controls	USA: Nine cities	1969–1985
Kabat 2013 [81]	Population-based cohort	493,188 adults aged 50–71 years at entry (NIH- AARP Diet and Health Study)	USA	1995–2006)
Kane 1999 [82]	Population-based case-control	695 AML cases 1374 controls	England	1991–1996
Kasim 2005 [83]	Population-based case-control	307 AML cases; 169 CML cases 5039 controls	Canada	1994–1997
Kroll 2012 [84]	Population-based cohort	1.3 million middle-aged women (UK Million Women cohort)	UK	1996–2009
Leal 2014 [85]	Population-based cohort	27,370 women aged 55–69 yrs. at entry (Iowa Women’s Health Study cohort)	USA: Iowa	1993–2004
Linet 1991 [86]	Population-based cohort	17,633 members of Lutheran Brotherhood	USA	1966–1986
Lv, 2011 [87]	Hospital-based case-control	403 MDS cases, 806 controls	China: Shanghai	2003–2006
Ma 2009; 2010 [88, 89]	Population-based cohort study	471,799 adults aged 50–71 years at entry (NIH AARP Diet and Health Study)	USA	1995–2003
Mele 1994 [90]	Hospital based case-control	55 MDS cases; 118 AML cases; 78 CML cases 467 controls	Italy	1986–1990
Mills 1990 [91]	Population-based cohort	34,000 Seventh Day Adventists	USA	1974–1982
Musselman 2013 [92]	Population-based case-control	413 AML cases; 184 CML cases 1022 controls	USA: Minnesota	2005–2009
Nagata 1999 [93]	Population-based case-control	111 MDS cases 830 controls	Japan	1995–1996
Nisse 2001 [94]	Population-based case-control	204 MDS cases 204 controls	Northern France	1991–1996
Parodi 2017 [95]	Population-based case-control	223 AML cases; 106 CML cases 1774 controls	Italy	1990–1993
Pasqualetti 1997 [96]	Hospital-based case-control	73 ANNL cases; 85 MDS cases; 92 MPN cases (includes 69 with CML)	Italy	circa 1971–1996
Pedersen 2018 [97]	Population-based cohort study	75, 896 adults in Denmark (Danish Health Examination Survey)	Denmark	2007–2015
Pekmezovic 2006 [98]	Hospital-based case-control	80 MDS cases 160 controls	Serbia Montenegro	2000–2003
Pogoda 2002 [99]	Population-based case-control	412 AML cases 412 controls	USA; Los Angeles	1987–1994
Richardson 2008 [100]	Population-based case-control	120 ANLL cases; 69 CML cases 423 controls	Germany	1986–1998
Sandler 1993 [101]	Population-based case-control	15 MDS cases; 423 AML cases 618 Controls	USA and Canada	1986–1989
Severson 1990 [102]	Population-based case-control	106 ANL cases (93 AML) 128 controls	USA: Seattle	1984–1986
Speer 2002 [103]	Registry-based case-control	604 AML cases 7112 controls (colon cancer patients)	USA: Orange County, California	1984–1993
Stagnaro 2001 [104]	Population-based case-control	105 AML cases; 105 CML cases 1765 controls	Italy	1990–1993
Strom 2005 [60]	Hospital-based case-control	354 MDS cases 452 controls	Texas	1999–2003
Strom 2012 [105]	Population-based case-control	638 AML cases 636 controls	Texas	2003–2007
Ugai 2017a;2017b [106, 107]	Population-based cohort	96,992 members of Japan Public Health Center-Based Prospective Study	Japan	1990–2012
Wakabayashi 1994 [108]	Hospital-based case-control	75 ANNL cases 150 controls	Japan	1981–1988
West 1995 [109]	Population-based case-control	400 MDS cases 400 controls	England and Wales	Not reported
Wong 2009 [110]	Hospital-based case-control study	722 AML cases 144 controls	China:Shanghai	2003–2007
Xu 2007 [111]	Population-based cohort	24,539 members of Three Mile Island cohort	USA; Pennsylvania	1979–1995

**Table 8:** Results of studies on tobacco smoking and specific type of myeloid malignancies

	Exposure	MDS		AML		CML		MPN.		Myeloid l.	
		Point estimate	95% CI	Point estimate	95% CI	Point estimate	95% CI	Point estimate	95% CI	Point estimate	95% CI
Averginou 2017 [70]	CS	0.94	0.46–1.91								
	ES	1.18	0.69–2.02								
Batty 2008 [71]	CS									5.08	1.78–14.5
	10 cpd_c									0.45	0.28–0.73
	10 yr_c									1.27	0.71–2.26
Bjork 2000 [72]	Recent smokers	2.0	1.3–3.1								
	> 10 cpd, > 40 yrs	3.5	1.9–6.4								
	> 40 pyrs	3.0	1.6–5.8								
Bjork 2001b [73]	> 10 cpd, > 20 yrs			1.6	1.0–2.4						
Bjork 2009 [74]	CS smoker	1.2	0.70–2.2	0.97	0.58–1.6						
	≥20 yrs	1.4	0.78–2.5	1.1	0.63–1.8						
	≥20 pyrs	1.6	0.85–3.1	1.1	0.60–2.0						
Brown 1992 [75]	CS			1.7	0.9–2.9	1.3	0.5–3.6				
	> 20 cpd			1.3	0.7–2.4	2.1	0.8–5.3				
	≥46 yrs			1.5	0.8–2.8	3.3	1.2–9.0				
Brownson 1991 [76]	≥20 cpd, males			1.2	0.7–1.9	0.8	0.4–1.6				
	≥20 cpd, females			1.4	0.8–2.2	0.5	0.2–1.4				
Dalamaga 2002 [77]	CS (non-drinker)	1.73	0.58–5.18								
Fernberg 2007 [78]	CS			1.50	1.06–2.11	0.69	0.42–1.14				
	> 20 cpd			1.59	0.9–2.79						
Ido 1996 [79]	ES	1.8	0.83–3.89								
Kabat 1988 [80]	ES			0.83	0.61–1.14	0.60	0.36–1.0				
	≥31 cpd			0.71	0.37–1.37						
Kabat 2013 [81]	≥ 21 cpd					1.53	1.03–2.27				
Kane 1999 [82]	CS			1.4	1.1–1.8						
	40+ yrs			1.3	0.9–1.9						
Kasim 2005 [83]	CS			1.4	1.1–1.8	0.7	0.4–1.1				
	> 20 pys			1.5	1.1–2.0	0.9	0.4–1.6				
Kroll 2012 [84]	≥ 15 cpd	1.98	1.67–2.35	1.08	0.80–1.46					1.69	1.46–1.96
Leal, 2014 [85]	CS							1.61	1.06–2.45		
Linet 1991 [86]	ES									0.8	0.3–1.8
	20+ cpd									1.3	0.5–3.8
Lv 2011 [87]	CS	1.37	0.89–2.12								
	≥20 yrs	1.22	0.91–1.63								
	≥20 cpd	1.06	0.75–1.50								
	≥20 pyrs	1.06	0.73–1.55								
Ma 2009; 2010 [88, 89]	CS	3.17	2.02–4.98	NR	NR						
	CS, > 1 ppd	4.70	2.68–8.24	2.29	1.38–3.79						
Mele 1994 [90]	> 20 pys	2.4	1.0–5.8	1.7	0.9–3.0	1.0	0.5–2.1				
	CS	1.2	0.4–3.3	1.6	0.9–2.8	1.3	0.7–2.6				
Mills 1990 [91]	CS									2.04	0.25–16.65
	> 25 cpd									3.55	1.14–11.07
	> 15 yrs									2.69	0.94–7.72
Musselman 2013 [92]	> 20 cpd			2.06	1.29–3.28	1.58	0.84–2.98				
Nagata 1999 [93]	CS	0.94	0.50–1.78								
	≥20 cpd	0.98	0.51–1.88								
	≥20 yrs	0.81	0.41–1.64								
Nisse 2001 [94]	CS/Ex-S	3.4	1.7–7.3								
	≥20 pyrs	2.6	1.1–6.8								
Parodi 2017 [95]	CS			0.43	0.23–0.82	0.81	0.35–1.19			0.52	0.31–0.88
Pasqualetti 1997 [96]	Heavy smokers	3.00	1.09–8.25	3.20	1.17–8.73			1.25	0.59–2.67		
Pedersen 2018 [97]	Daily smokers							2.5	1.3–5.1		
Pekmezovic 2006 [98]	> 25 yrs	1.0	0.4–2.3								
	> 20 cpd	1.3	0.4–3.6								
Pogoda 2002 [99]	ES			1.2	0.9–1.6						
	> 35 yrs.^a^			2.9	1.1–7.2						
	> 20 cpd^a^			3.4	1.4–8.2						
	> 40 pyrs^a^			2.3	0.9–5.6						
Richardson 2008 [100]	CS					1.04	0.47–2.30				
	10 pys_c					1.07	0.97–1.17				
Sandler 1993 [101]	ES	1.64	0.53–5.07	1.18	0.91–1.54						
	> 40 pyrs			1.42	1.00–2.07						
Severson 1990 [102]	ES			2.1	1.2–3.9						
	CS			1.9	0.9–3.7						
	≥40 pyrs			3.1	1.4–7.4						
Speer 2002 [103]	CS			2.2	1.6–3.0						
Stagnaro 2001 [104]	CS			0.93	0.61–1.4	0.59	0.34–1.0				
	≥20 cpd			1.2	0.74–1.9	0.45	0.23–0.87				
	≥37 yrs			1.2	0.72–1.9	0.56	0.30–1.1				
Strom 2005 [60]	ES	1.65	1.19–2.30								
	Per py	1.01	1.005–1.02								
Strom 2012 [105]	> 30 pyrs, women			1.34	0.72–2.50						
	> 30 pyrs, men			1.86	1.15–3.02						
Ugai 2017a [106]	> 30 pys/CS			2.02	1.00–4.06	1.19	0.36–3.91				
Ugai 2017b [107]	CS	1.62	0.80–3.27								
	> 30 pyrs	1.74	0.84–3.59								
Wakabayaski 1994 [108]	≥31 cpd			2.99	0.66–13.47						
West 1995 [109]	ES	1.16	0.83–1.63								
	≥25 cpd	1.0	(NR)								
Wong 2009 [110]	> 20 cpd	0.96	0.58–1.57								
	> 20 yrs	1.33	0.99–1.77								
	> 20 pyrs	1.29	0.95–1.75								
Xu 2007 [111]	CS			3.47	1.00–12.0						
	> 20 cpd			1.07	0.23–5.01						
	> 30 yrs			3.64	0.80–16.5						
	> 20 pys			1.11	0.19–6.36						

^a^ results for FAB subtype M2, 107 cases/107 controls

Abbreviations: *CS* Current smoker; *cpd* Cigarettes per day; *ES* Ever smoked (combined current and former smokers); *pys* Pack-years; *RR* Relative risk; *yrs*. Years of smoking

One of the largest studies to examine leukemia risks among smokers was a prospective cohort of 1.3 million middle-aged women recruited for breast cancer screening during 1996–2001 and followed for mortality through 2009 [84]. The investigators identified death due to myeloid neoplasm in 831 cohort members and reported a statistically significantly increased risk (RR = 1.33, 95% CI 1.24–1.42). The increase was driven by a significant RR for MPN/MDS (RR = 1.42, 95% CI 1.31–1.55), whereas the RR for AML was not elevated (RR = 1.10, 95% CI 0.96–1.26). Relative risks also increased with increased intensity of smoking for myeloproliferative/myelodysplastic disease, but not for AML [84].

A cohort study of over 330,000 Swedish construction workers with follow-up for mortality through 2004 reported a statistically significant association between “current” smoking and AML risk (RR = 1.50, 95%CI: 1.06, 2.11), but no association with CML (RR = 0.69, 95% CI 0.42–1.14). For AML, relative risks did not increase with increasing intensity of smoking [78].

Table 9 presents summary risk estimates for myeloid malignancies by various smoking exposure metrics and study type. Several meta-analyses demonstrated significant heterogeneity, as indicated by the high I^2^ statistic and associated low *p*-values. However, publication bias generally was not indicated.

**Table 9:** Meta-analyses of studies specific leukemia types for smoking

Characteristics	No. of estimates	Meta-RR estimate (95%CI)	I^2^ test (%)	p-value	Egger’s test
**ML**					
Overall	6	1.54 (0.79–3.01)	81.80	0.2038	0.8539
Smoking status					
Current smoker	3	1.55 (0.44–5.46)	75.24	0.4942	0.4930
Ever smoker	3	1.68 (1.45–1.94)	0.00	< 0.0001	0.9406
Study type					
Case-control studies	1	0.52 (0.31–0.88)	000	< 0.00001	NA
Cohort studies	5	1.71 (1.49–1.98)	0.00	< 0..0001	0.5972
**AML**					
Overall	28	1.43 (1.25–1.62)	56.25	< 0.0001	0.0848
Smoking status					
Current smoker	21	1.49 (1.28–1.72)	53.64	< 0.0001	0.3747
Ever smoker	7	1.22 (1.00–1.48)	34.43	0.00497	0.1008
Study type					
Case-control studies	23	1.43 (1.26–1.63)	46.25	< 0.0001	0.3784
Cohort studies	5	1.43 (1.03–1.99)	69.14	0.0351	0.0882
**CML**					
Overall	14	0.93 (0.74–1.16)	40.44	0.5242	0.7871
Smoking status					
Current smoker	8	0.81 (0.64–1.01)	0.00	0.0637	0.0347^a^
Ever smoker	6	1.05 (0.71–1.56)	56.51	0.8101	0.6791
Study type					
Case-control studies	11	0.88 (0.69–1.11)	25.34	0.2641	0.1266
Cohort studies	3	1.08 (0.67–1.74)	48.86	0.7513	0.8573
**MDS**					
Overall	22	1.66 (1.38–2.00)	61.89	< 0.0001	0.7014
Smoking status					
Current smoker	13	1.69 (1.28–2.22)	62.94	0.0002	0.9380
Ever smoker	7	1.51 (1.21–1.87)	44.07	0.0002	0.1445
Study type					
Case-control studies	18	1.42 (1.25–1.62)	2.32	< 0.0001	0.3969
Cohort studies	4	2.58 (1.80–3.70)	63.99	< 0.0001	0.4004
**MPN**					
Overall	3	1.70 (1.23–2.34)	0.00	0.0013	0.9210
Smoking status					
Current smoker	3	1.70 (1.23–2.34)	0.00	0.0013	0.9210
Ever smoker	0				NA
Study type					
Case-control studies	1	1.25 (0.59–2.66)	0.00	0.5623	NA
Cohort studies	2	1.82 (1.27–2.59)	0.00	0.0011	NA

^a^statistically significant

The meta-analysis for smoking and AML was based on 28 studies and resulted in a summary RR of 1.43 (95% CI 1.25–1.62; I^2^ = 56.25%) with slightly higher meta-RR for current smokers and a lower meta-RR for ever smokers. Meta-analysis of results for CML was based on 14 studies, generating a summary RR of 0.93 (95% CI 0.74–1.16; I^2^ = 40.44%) with similar results for current and ever smokers. The meta-RR for myeloid leukemias combined (i.e., based on eight studies not differentiating by leukemia type) was 1.54 (95% CI 0.79–3.01; I^2^ = 81.80%) with similar results for current and ever smokers. The meta-analysis of overall results on MDS was based on 22 studies and resulted in a summary RR of 1.66 (95% CI 1.38–2.00; I^2^ = 61.89%) with similar results for current and ever smokers. Only three studies were identified for MPN resulting in a summary RR of 1.70 (95% CI 1.23–2.34; I^2^ = 0%) for current smokers*.* Publication bias appears unlikely for the meta-analyses on smoking except for the Egger’s test results for the category of current smoker and CML. Visual inspection of the funnel plot, however, did not suggest any publication bias (see supplemental file).

In contrast to benzene, the evidence on the risk of specific leukemia subtypes from tobacco smoking indicates an association with AML, but not with CML. Similar to benzene, there is evidence of an increased risk of MDS. Although only three studies were identified, risk of myeloproliferative/myelodysplastic neoplasm (MPN) appears to be increased among smokers.

## Discussion

The myeloid malignancies clearly have different clinical features and characteristic genetic aberrations and therefore, they should be evaluated separately in epidemiological studies intended to identify risk factors and potential causes.

We found little consistency in the way leukemias were evaluated, and they often were analyzed in aggregate, mixing myeloid and lymphocytic leukemias. The more recent benzene cohort studies were the exception, as they specifically evaluated AML, CML and MDS separately. Some analyses evaluated myeloid malignancies separately from the lymphocytic neoplasms, but still combined AML and CML, despite evidence of different mutations in genes and other risk factors that indicate different etiologies. Despite the determination that the epidemiological evidence was sufficient for purposes of establishing causation for leukemia, our review identified only small numbers of studies that actually reported results for specific types of myeloid neoplasms. Furthermore, where specific diseases were considered, small numbers of observed events often limited the precision of risk estimates.

For example, for butadiene, only one study analyzed risks by specific leukemia type, and findings were mixed: statistically significant associations were reported for CML among laboratory workers (based on only three deaths) but not for AML [25]. That results on butadiene exposure and myeloid malignancies are based on a single study and do not allow any causal conclusions does not necessarily mean that the IARC conclusion of “sufficient” human evidence is incorrect. Rather, it indicates that the relationship, if any, with one or more specific type of leukemia cannot be discerned based on available epidemiological evidence.

The updated meta-RRs for formaldehyde showed no consistent relationship with AML, CML or myeloid leukemia overall, confirming an earlier meta-analysis [28]. A further meta-analysis of the highest exposure groups was not conducted due to a lack of a common exposure metric. A very large registry-based linkage study demonstrated no increased risk and, in fact, reported a statistically significant deficit of incident AML cases among groups potentially occupationally exposed to formaldehyde [34]. Our findings suggest that the updated human evidence for formaldehyde and leukemia (of any type) may not be sufficient as determined by IARC and should be revisited. Combined with the lack of support from animal and mechanistic studies, it is unlikely that formaldehyde causes leukemia in humans [112].

A causal relationship between benzene exposure and AML has been recognized for decades and our meta-analyses indicate a significant increased risk overall and at high levels of exposure, yet the largest study, the Nordic registry study, demonstrated no association [34]. Other recent high-quality studies also indicate no clear association between benzene and AML risk [33, 42, 48, 55], possibly due to generally low exposure concentrations that do not exceed a possible exposure threshold for risk. The most comprehensive study on benzene and incident leukemia and myeloid neoplasm risks demonstrated a stronger association between benzene and risk of MDS than for AML, especially among workers with high peak exposures [58]. However, subsequent analyses of the Canadian sub-cohort were not consistent with these findings [68]. An association was reported between benzene and CML, but this was limited to case-control studies and may reflect potential reporting bias. Nevertheless, these findings epidemiologically underscore the importance of examining and contrasting results for specific malignancies (at least initially) and that exposure metric may play an important role in identifying causal associations [27].

Findings for tobacco smoking and myeloid leukemia were consistently positive for AML and negative for CML. The meta-RR for AML demonstrated a 50% statistically significantly increased risk, whether based on seven studies reporting individual leukemia types or the six in which AML and CML were reported separately. These results, and specifically the statistically significant positive meta-RR for AML and null findings for CML, further underscore the importance of examining epidemiologically narrowly defined or disease-specific relationships.

An ancillary finding of this evaluation is the surprisingly limited body of epidemiological studies aimed at addressing and differentiating risks by specific types of myeloid malignancy. While observing small numbers of any specific leukemia will plague all but the largest studies (or studies in which a strong association is indicated), we would argue that arbitrarily combining possibly discrete disease entities to improve “statistical power” will not help elucidate their specific causes; rather, this technique likely will dilute any true malignancy-specific associations and may lead to erroneous conclusions. One exception might be the subset of MDS cases that progresses to AML: these may reflect different clinical stages in the progression of the same disease. Nevertheless, publishing results based even on small numbers will facilitate combining results across studies using meta-analysis. It is conceivable that apparently negative findings based on small numbers may not be published, leading to potential “small numbers” or “negative study” publication bias, of which we found some evidence. While concerns of uncontrolled confounding arise in many occupational epidemiological settings, it is unlikely to be problematic in this context, primarily because there are no common exposures or risk factors that are strongly associated with all (or even multiple) types of leukemia. Progress in understanding the genetic factors underlying each of the myeloid neoplasms likely will guide future epidemiological studies to improve their ability to define appropriate combinations of myeloid malignancies and to isolate environmental risk factors that may be among their causes.

Nevertheless, our detailed evaluation of the four environmental chemical agents summarized here highlights important differences in risks by myeloid malignancies and provides support for reporting disease-specific findings from studies of environmental agents and risk of specific myeloid leukemias or other LHM. They also build on clinical observations that treatments with chemotherapy drugs lead to high incidence of AML and MDS (and possibly ALL) and that the genetic changes in therapy-related myeloid neoplasm reflect some specificity for the type of chemotherapy administered, but that chemotherapy does not lead to appreciable increases in CML, MPN, or lymphoid malignancies [2]. Meanwhile, epidemiological findings based on small numbers of specific LHM should be reported but appropriately caveated and not over-interpreted, as these results statistically will be unstable with likely false-positive and perhaps more likely false-negative relative risk estimates. Similarly, findings based on analyses of multiple types of leukemias and other LHM should be examined further and if possible, groups of LHM deconstructed, to identify the specific neoplasms that may be driving an observed association or situations where true associations may be diluted or masked.

## Supplementary Information

**Additional file 1.**

## Data Availability

All data generated or analysed during this study are derived from publicly available peer-reviewed scientific publications and are presented in this article and its supplemental file.

## Abbreviations

aCML: Atypical chronic myeloid leukemia
ALL: Acute lymphoblastic leukemia
AML: Acute myeloid leukemia
ANLL: Acute nonlymphocytic leukemkia
B-cells: B lymphocytes
CI: Confidence interval
CLL: Chronic lymphocytic leukemia
CML: Chronic myeloid leukemia
CMML: Chronic myelomonocytic leukemia
cpd: Cigarettes per day
HPC: Hematopoietic progenitor cells
IARC: International Agency for Research on Cancer
JMML: Juvenile myelomonocytic leukemia
LHM: Lymphohematopoietic malignancies
m-RR: Meta-relative risk
MDS: Myelodysplastic syndrome (MDS)
MPN: Myeloproliferative neoplasms
MDS/MPN: Myelodysplastic/myeloproliferative neoplasms
methyl-CCNU: semustine
MOPP: Vincristine-prednisone-nitrogen mustard-procarbazine
NHL: Non-Hodgkin lymphoma
NK cells: Natural killer cells
PMR: Proportionate mortality ratio
PPM: parts per million
PRISMA: Preferred reporting items for systematic reviews and meta-analyses
pyrs: Pack-years
RR: Relative risk
SBR: Styrene–butadiene rubber
SMR: Standardized mortality ratio
T-cells: T lymphocyte
WHO: World Health Organization.
