# Supplementary material for: The importance of evaluating specific myeloid malignancies in epidemiological studies of environmental carcinogens
Source: BMC Cancer. 2021 Mar 6;21:227. doi: 10.1186/s12885-021-07908-3 (PMC7936449; doi:10.1186/s12885-021-07908-3)

**Supplemental File:** Funnel plots of meta-analyses for benzene and tobacco smoking.

**Benzene - AML**

Overall


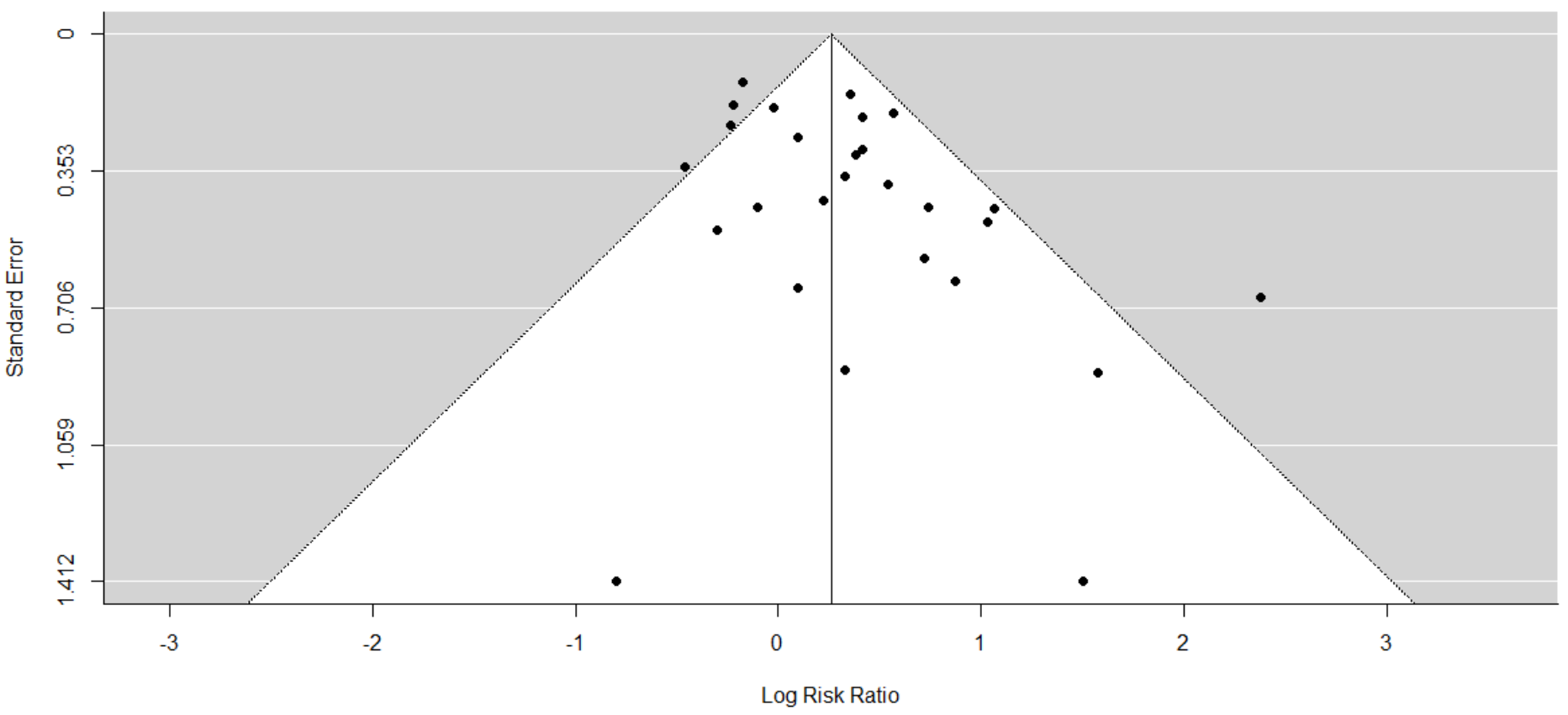


High exposure


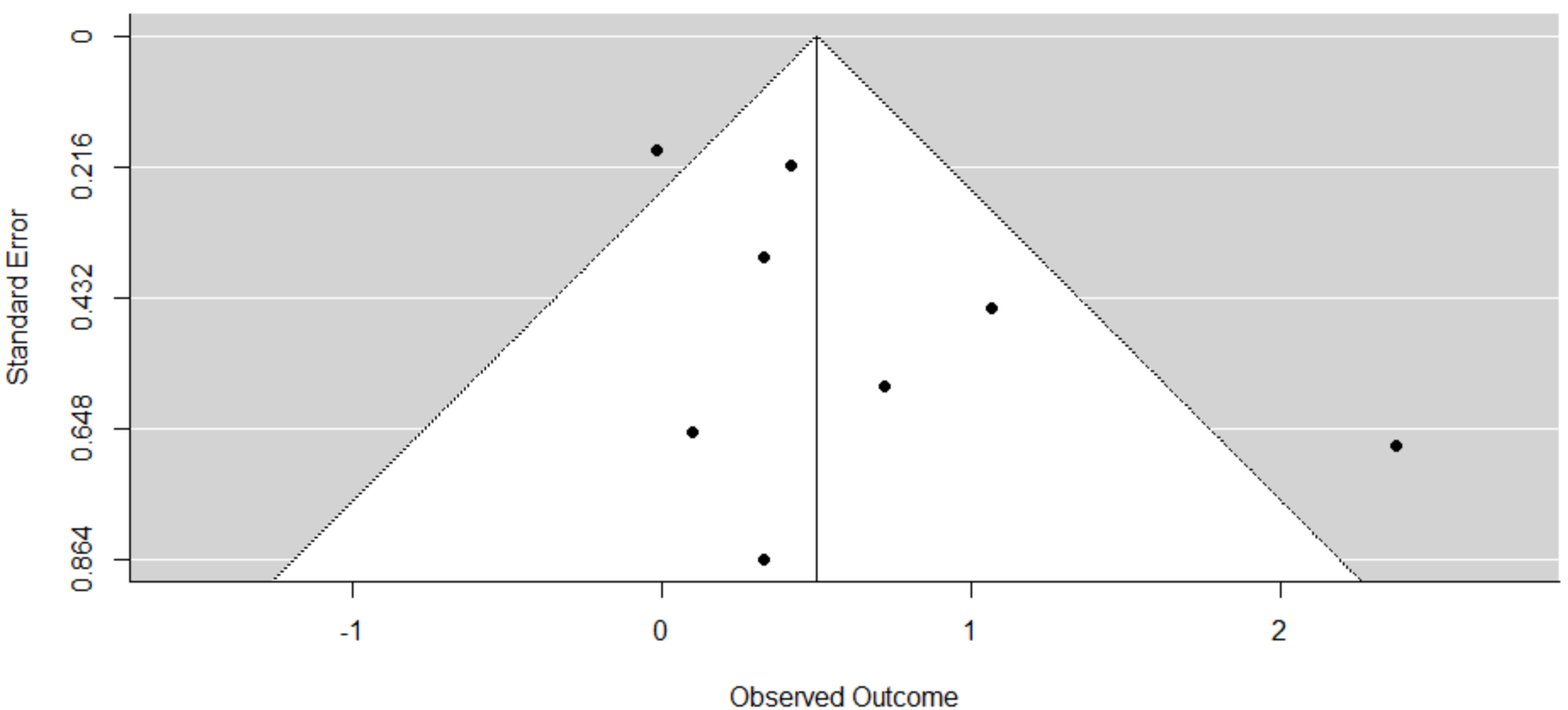


Low exposure


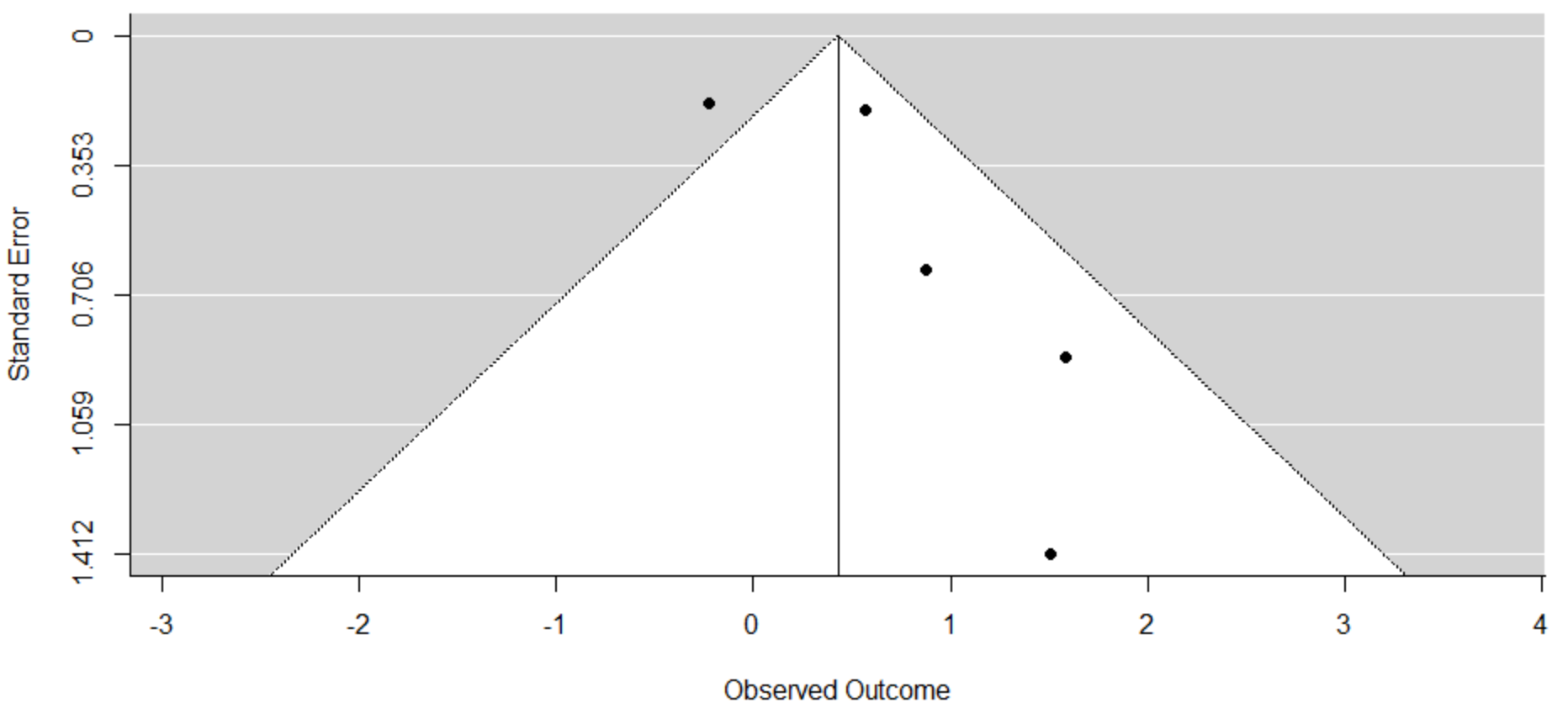


Any exposure


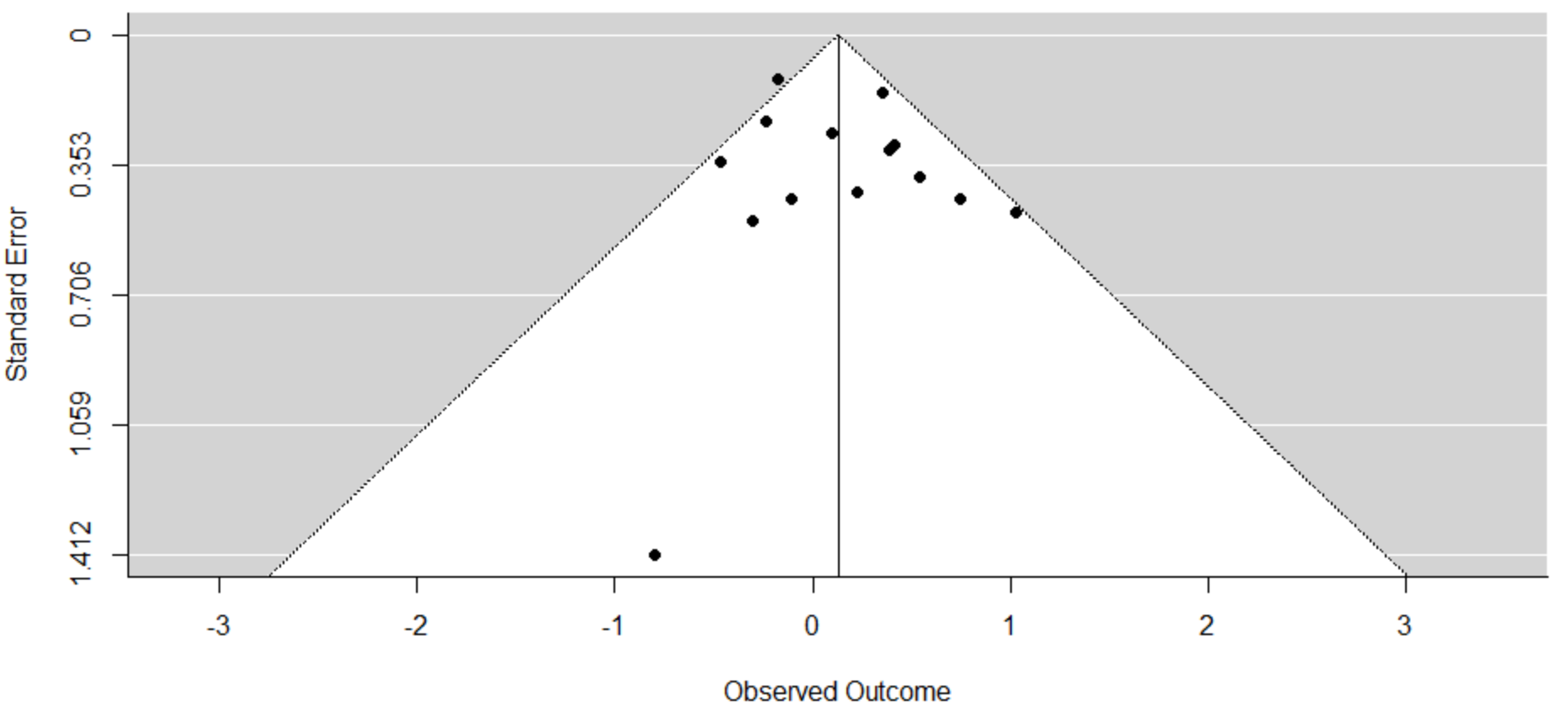


Case-control studies


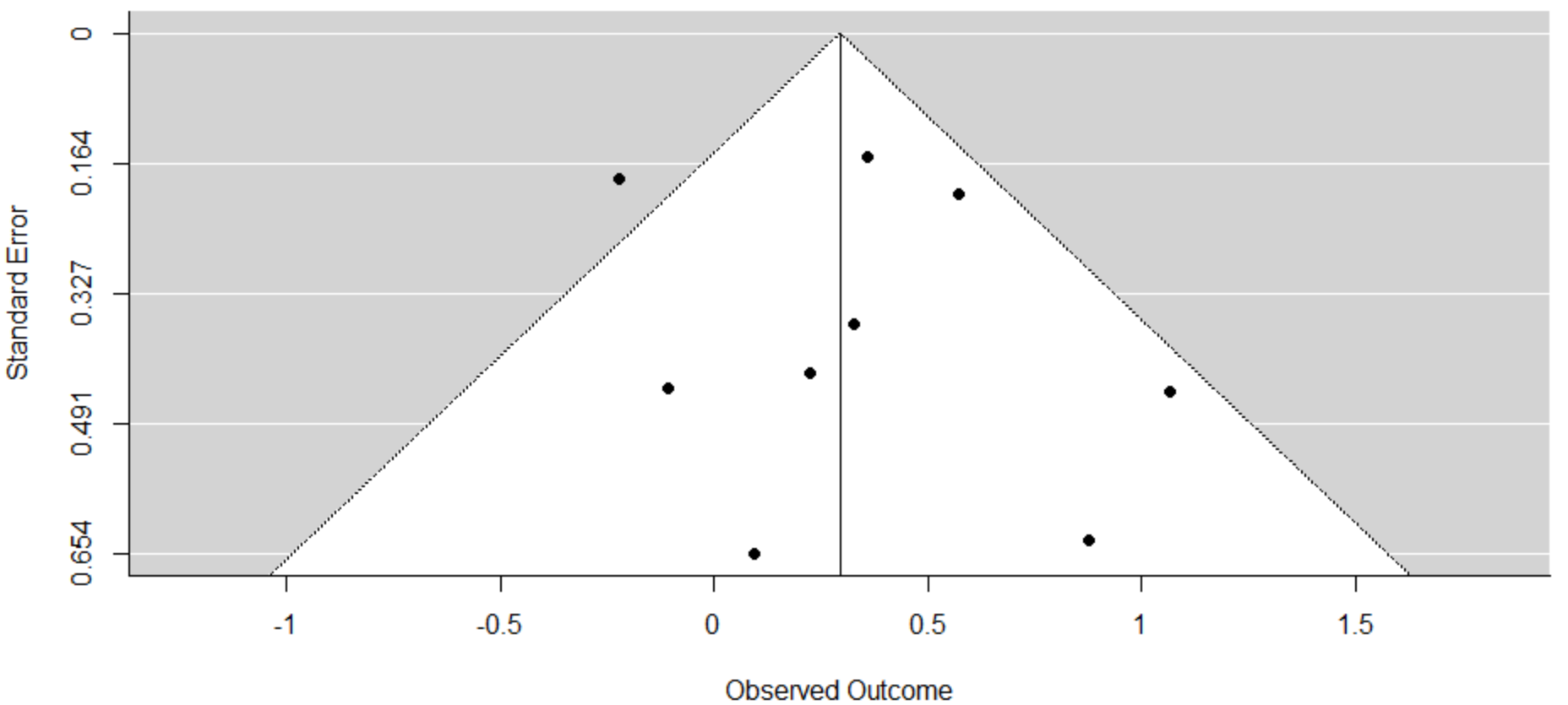


Cohort studies


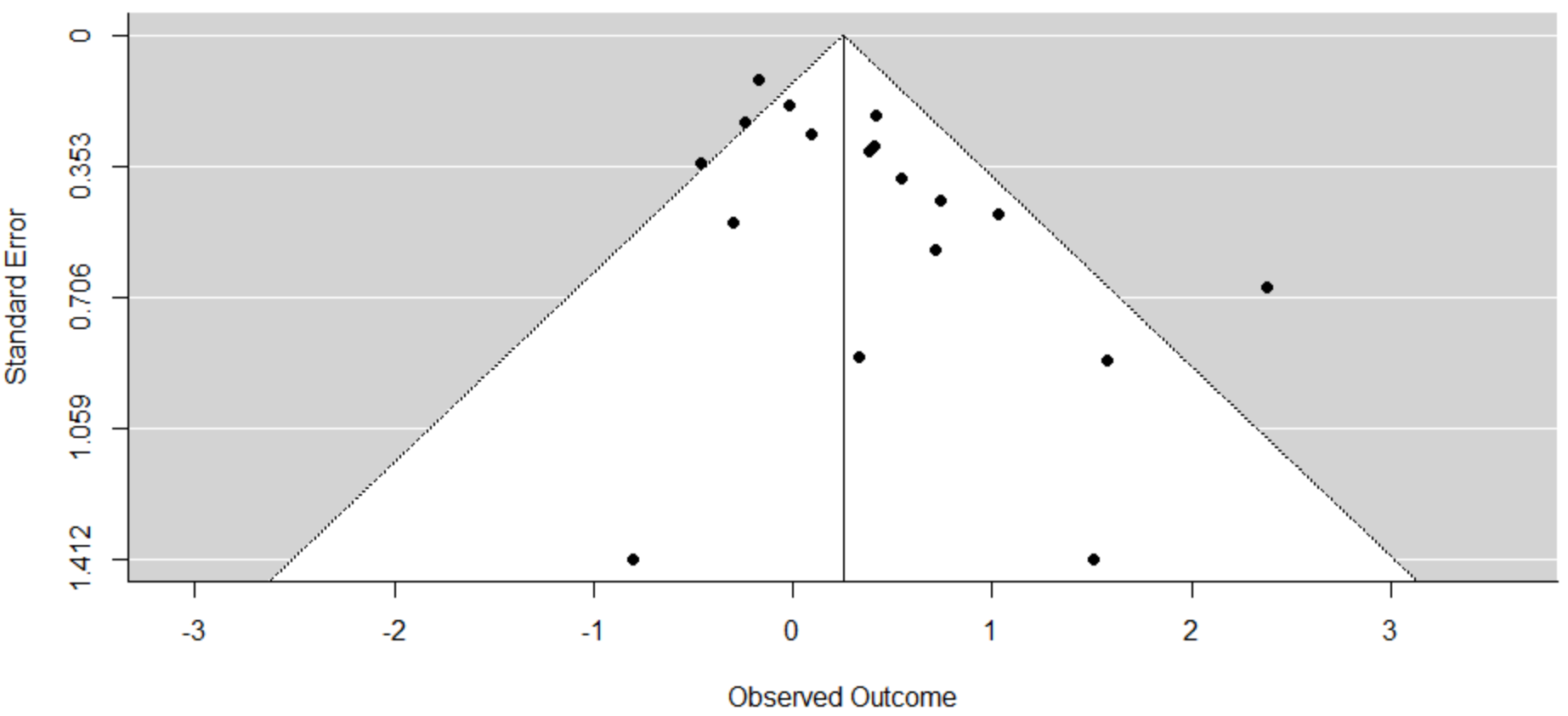


**Benzene - CML**

Overall


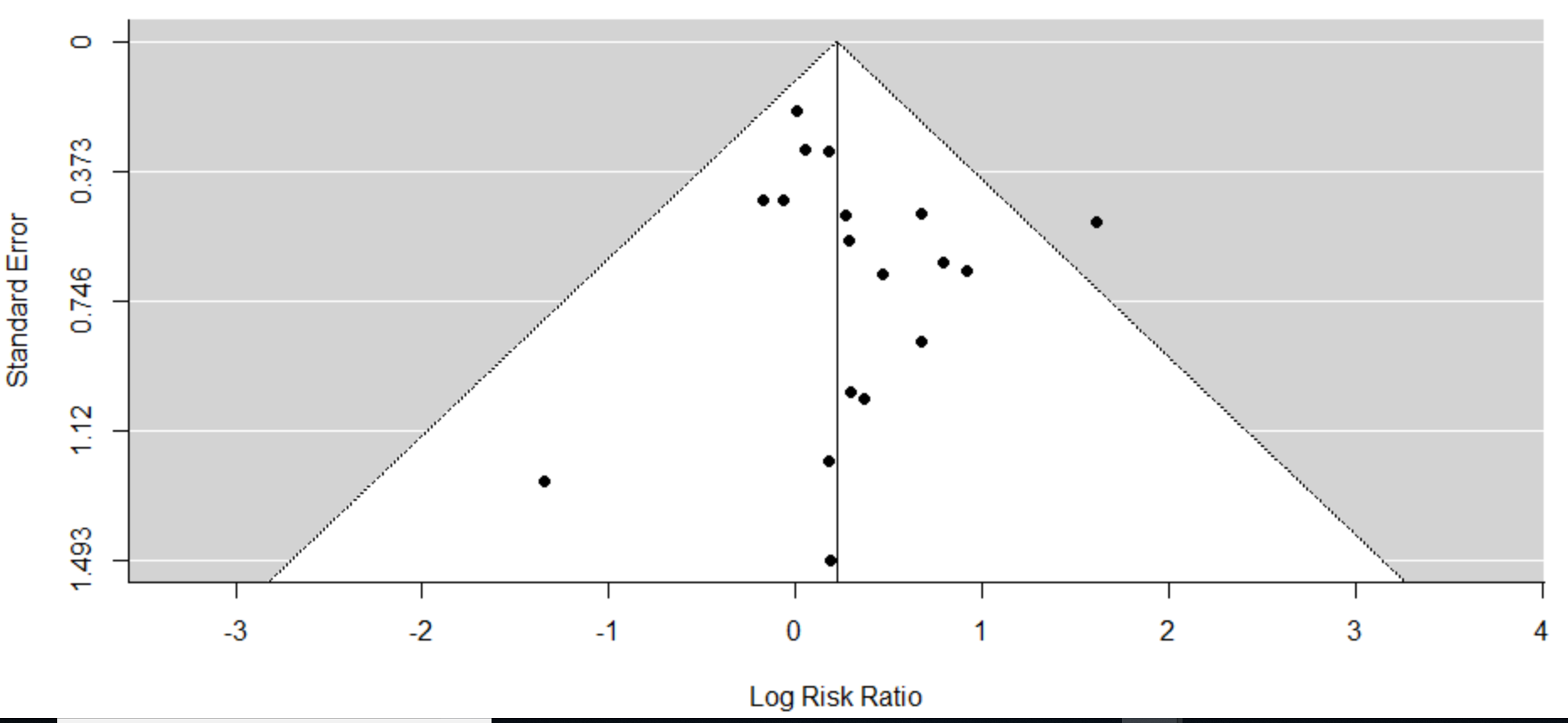


High exposure


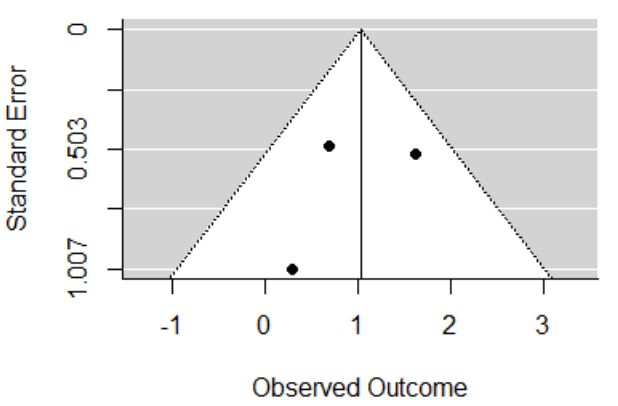


Low exposure


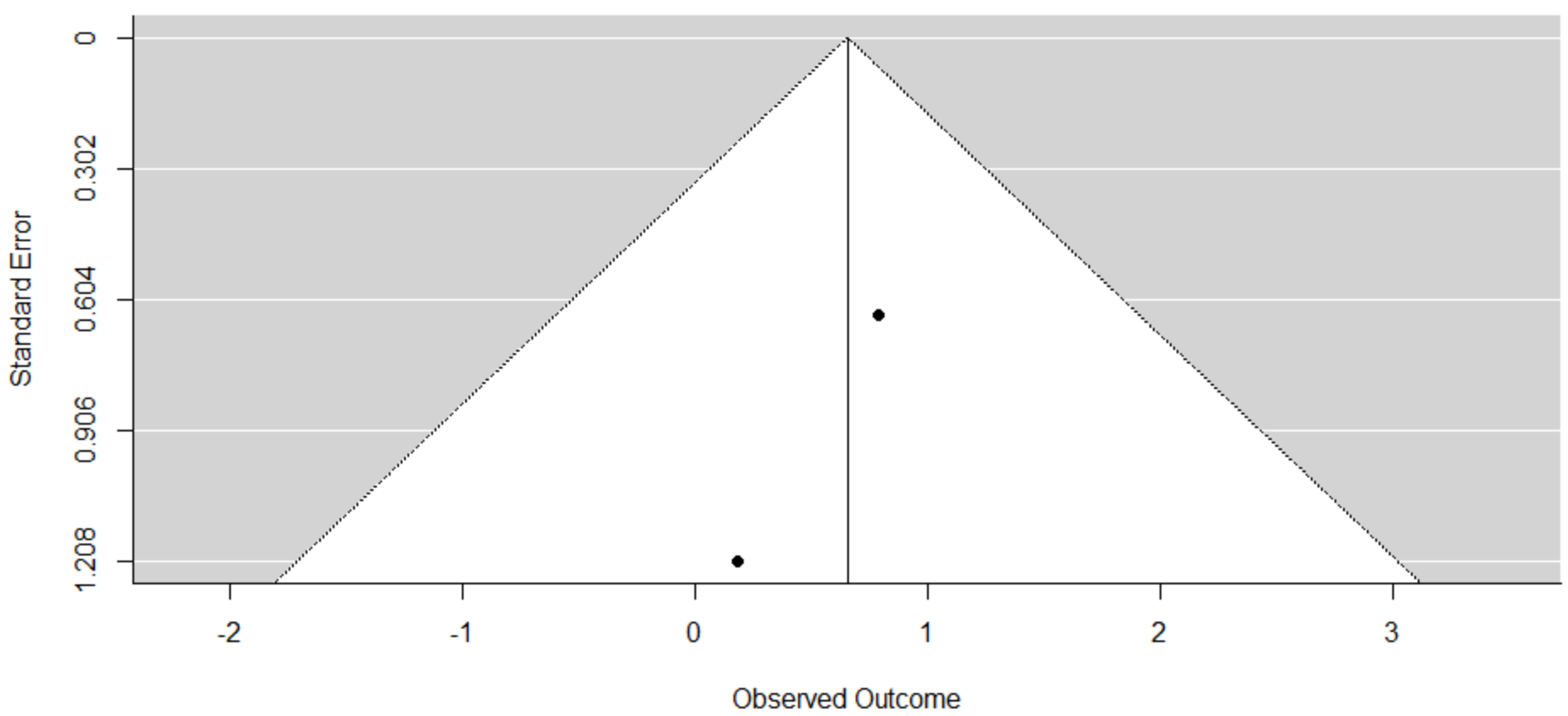


Any exposure


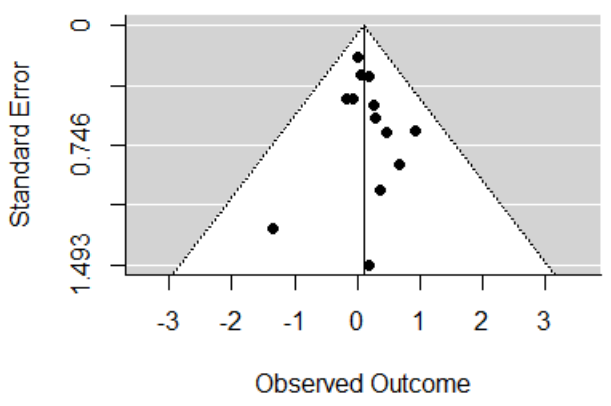


Case-control studies


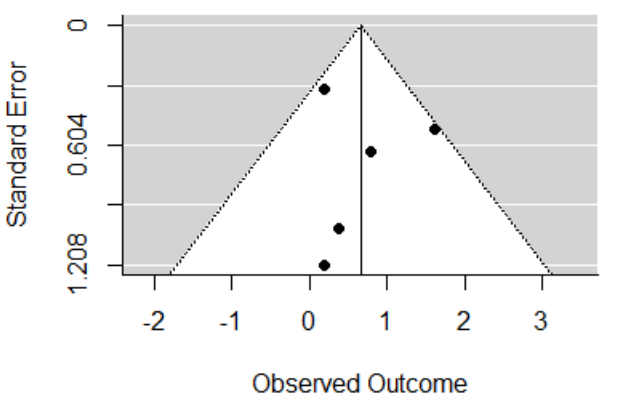


Cohort studies


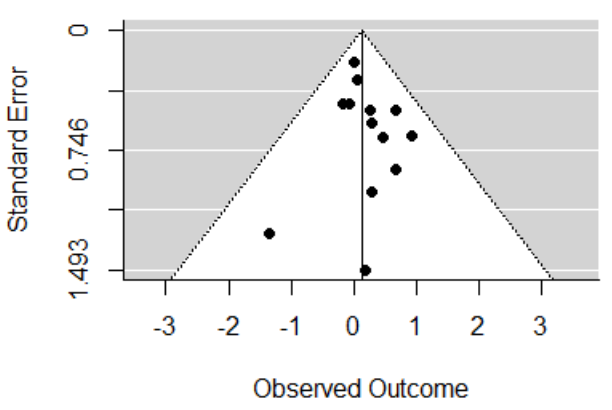


**Benzene – ML**

Overall


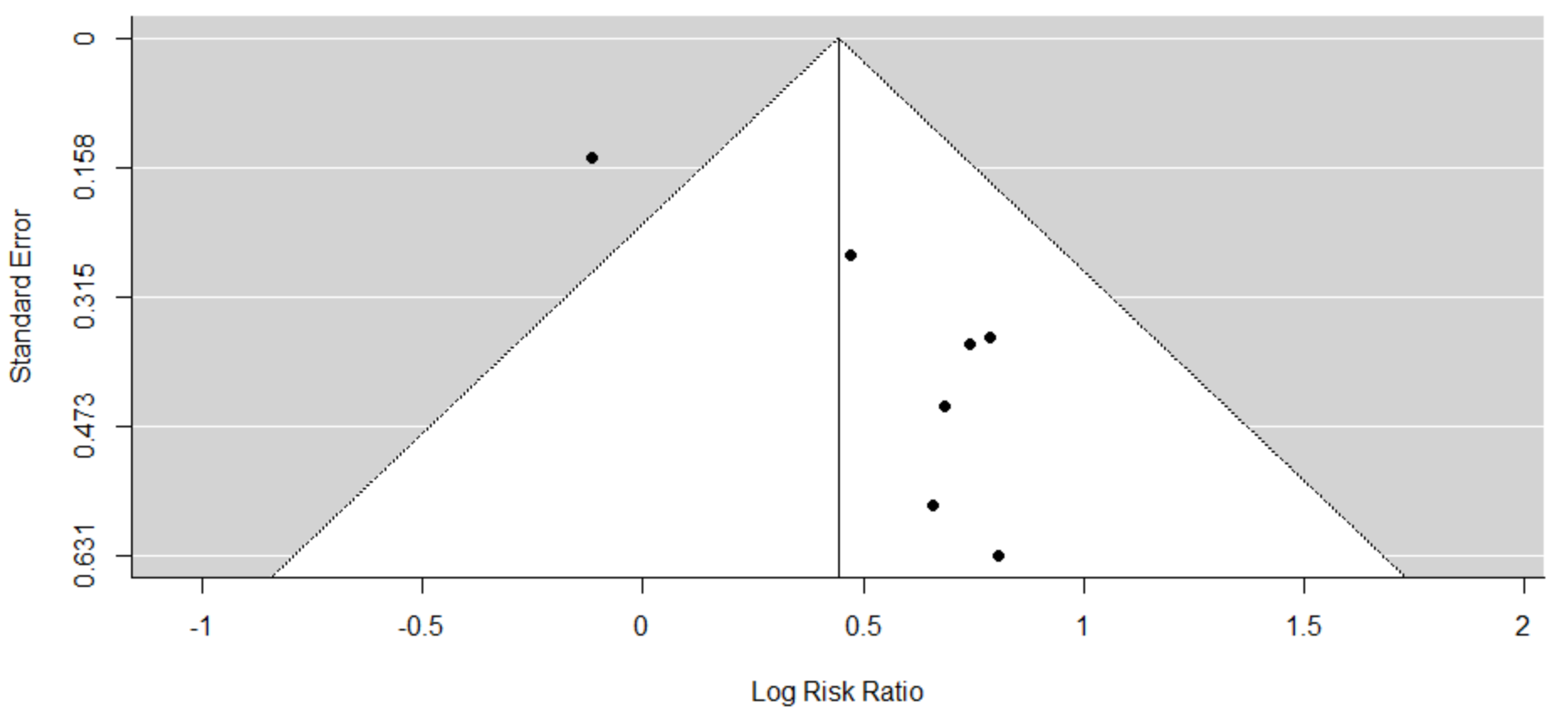


High exposure


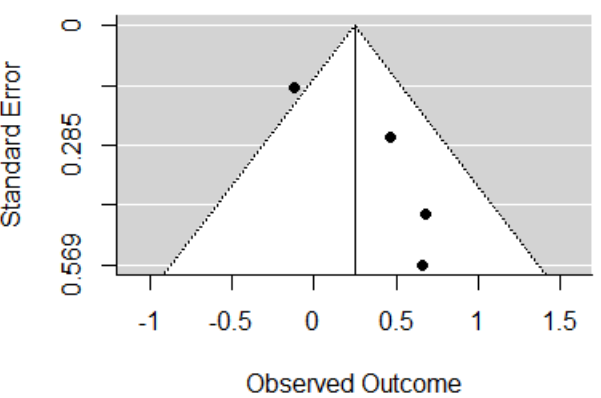


Cohort studies


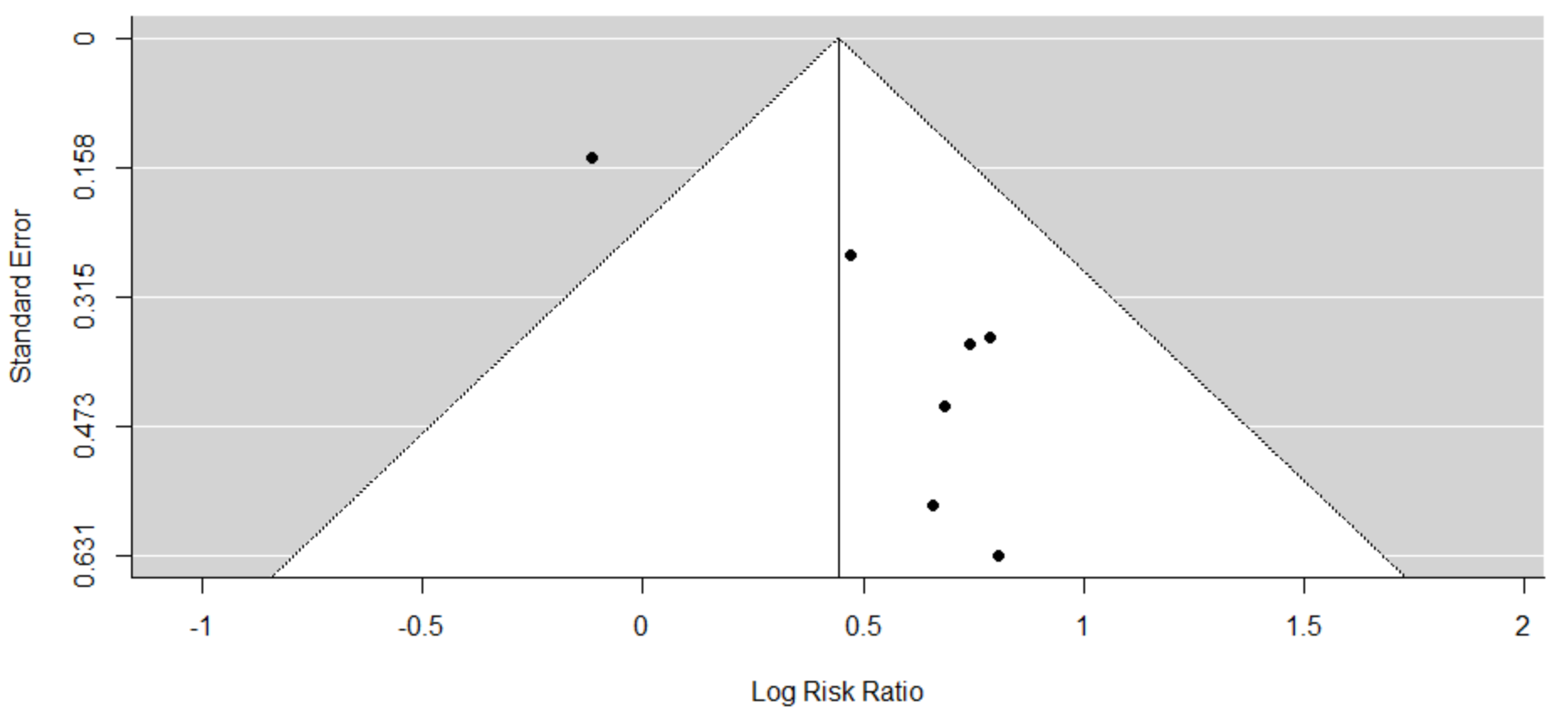


**Benzene - MDS**

Overall


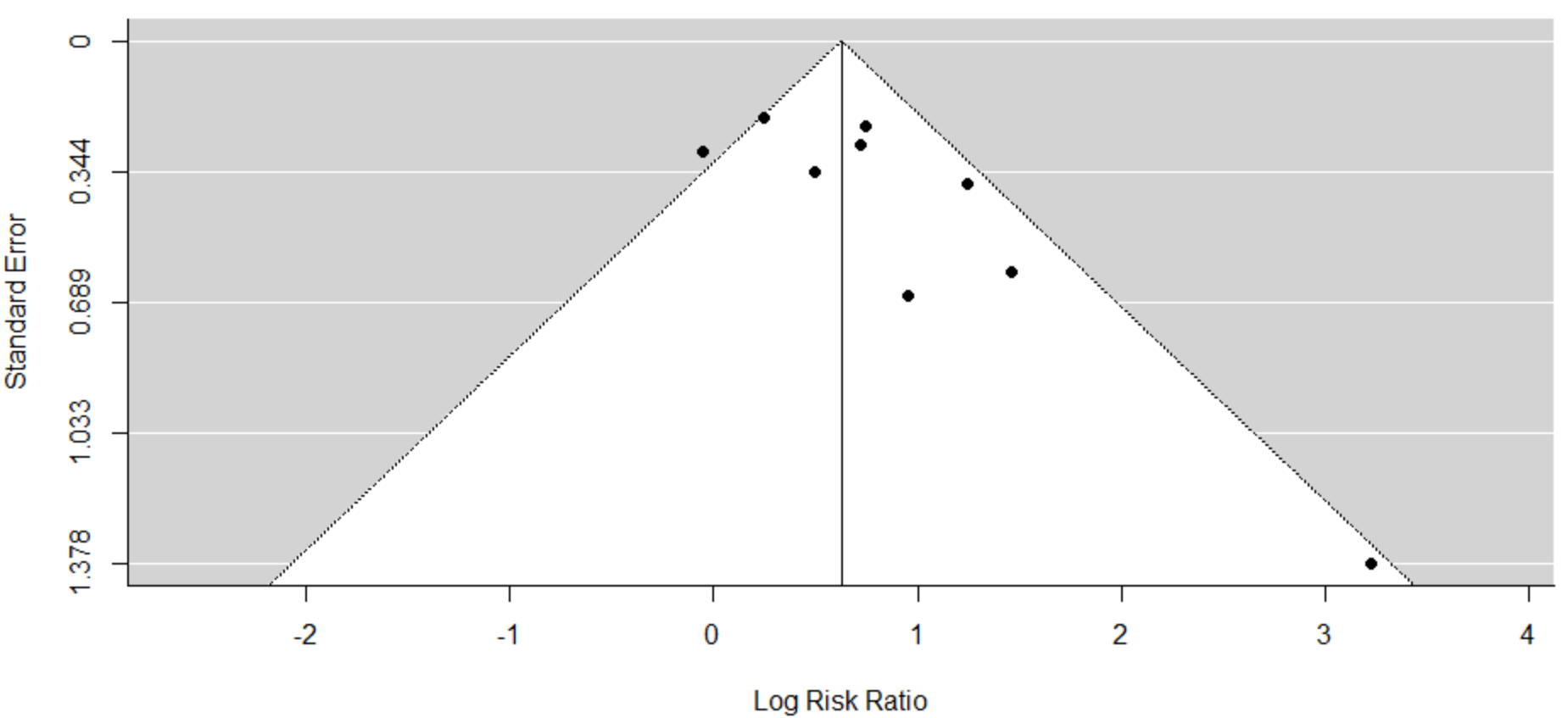


High exposure


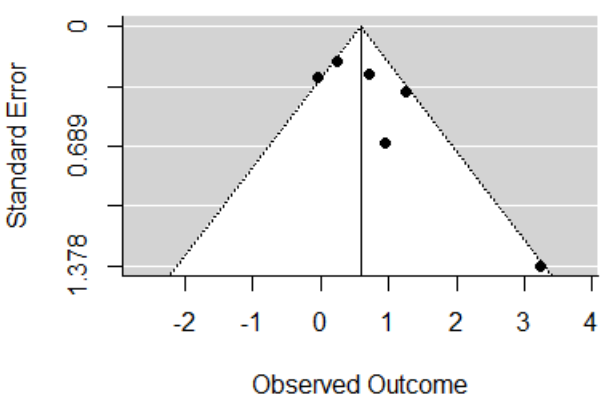


Case-control studies


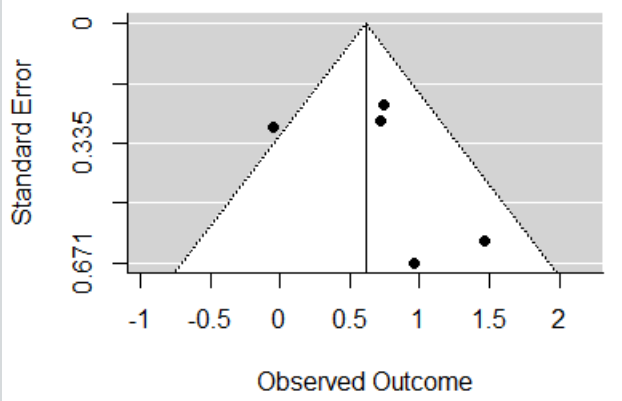


Cohort studies


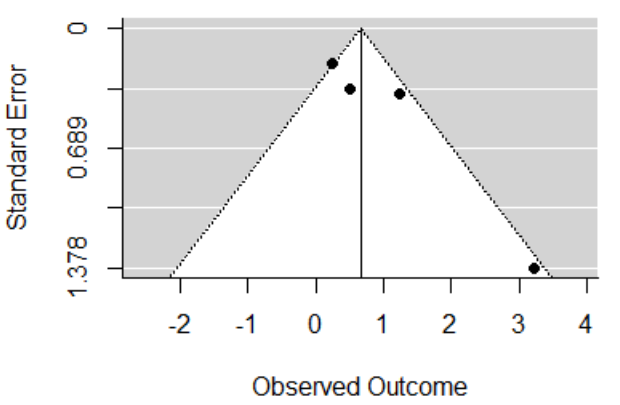


**Smoking – CML**

Overall


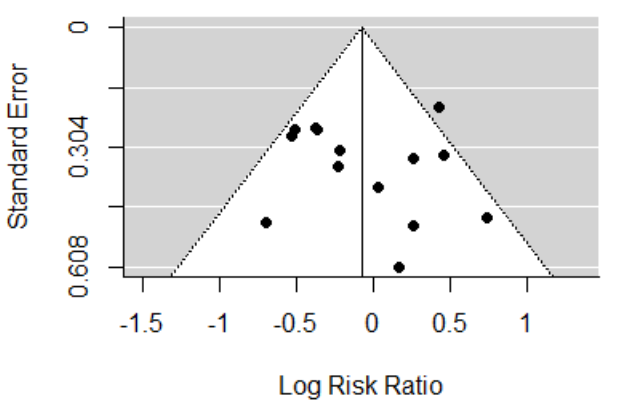


Current Smoker


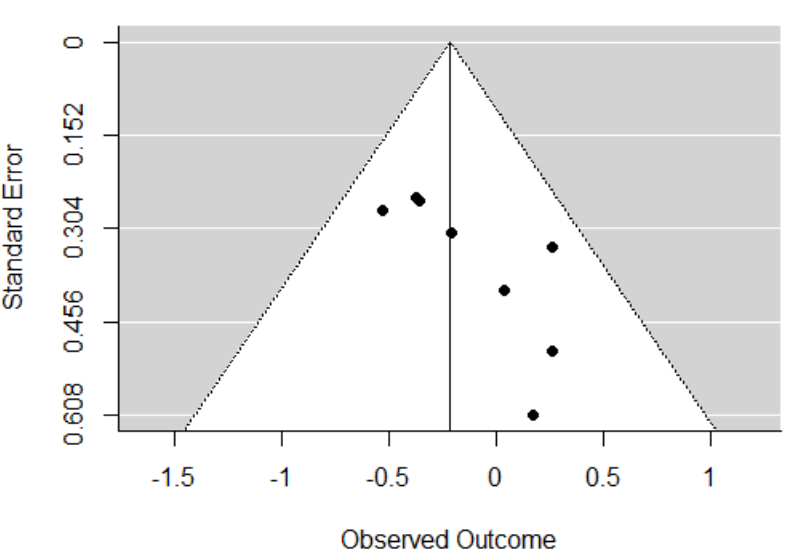


Ever Smoker


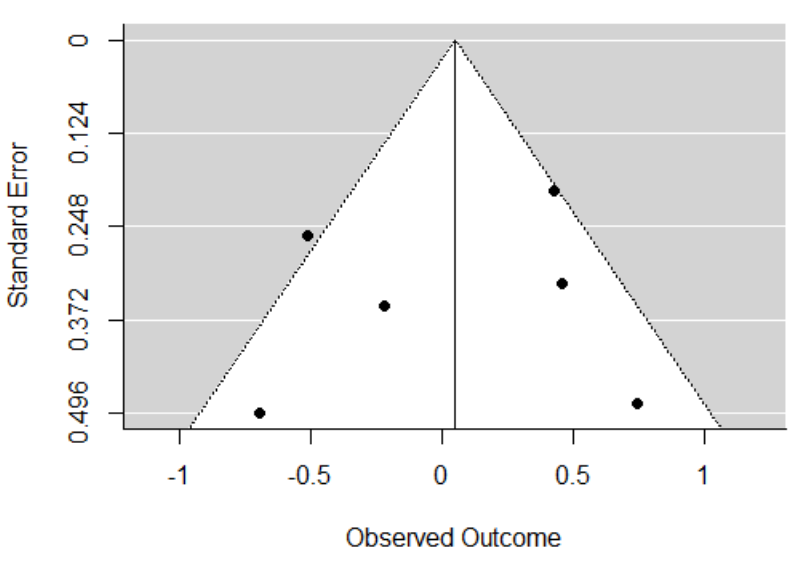


Case-control studies


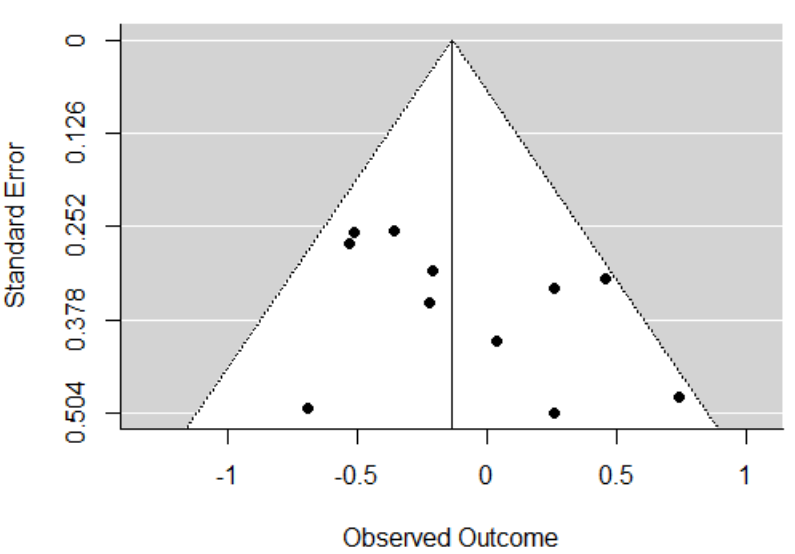


Cohort studies


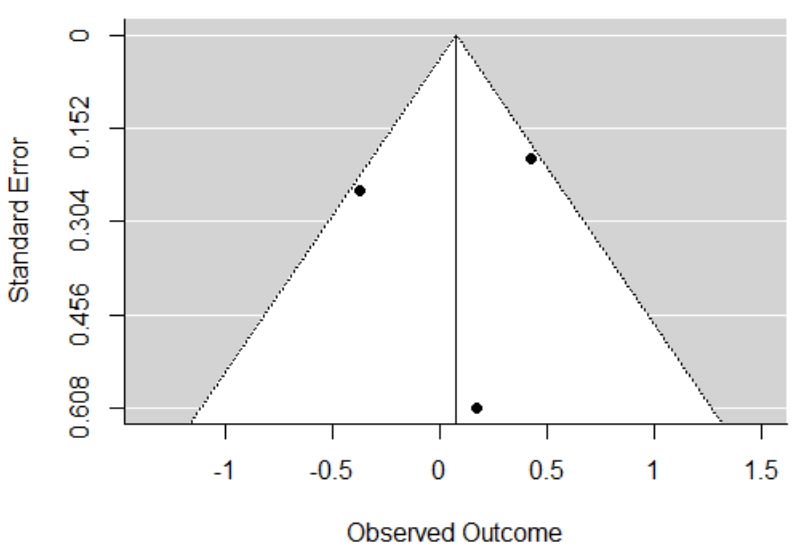


**Smoking – AML**

Overall


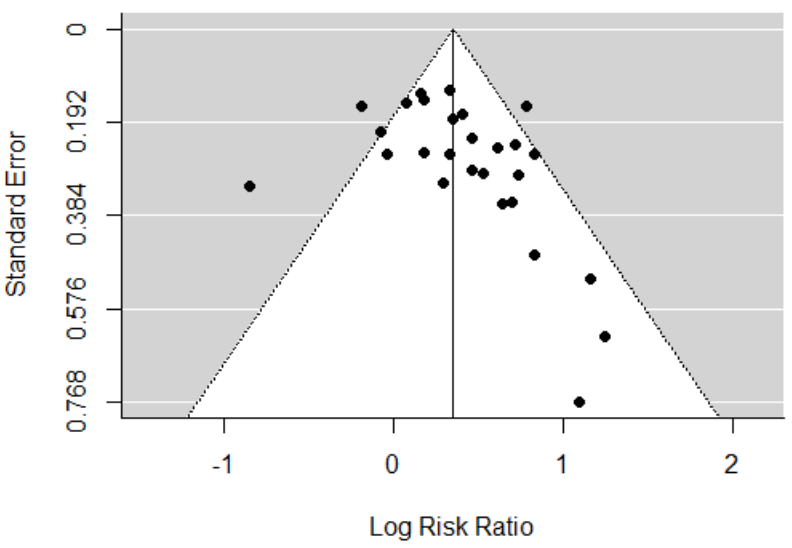


Current Smoker


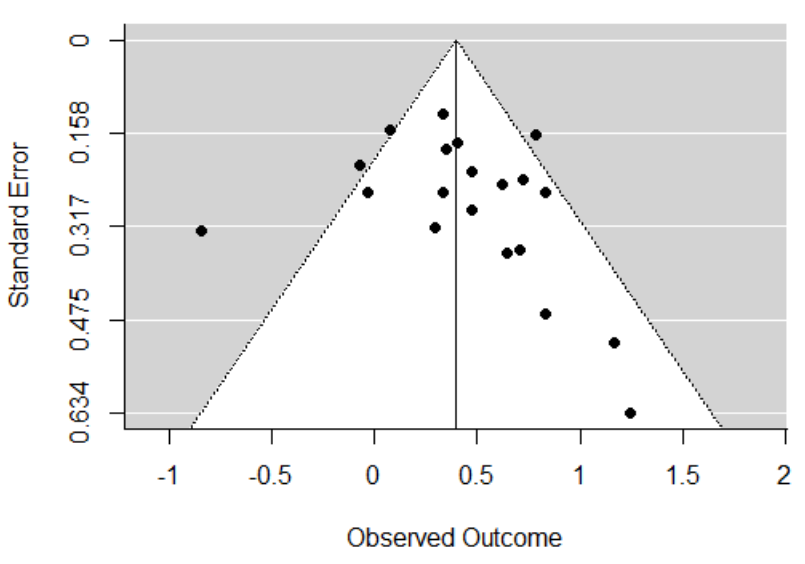


Ever Smoker

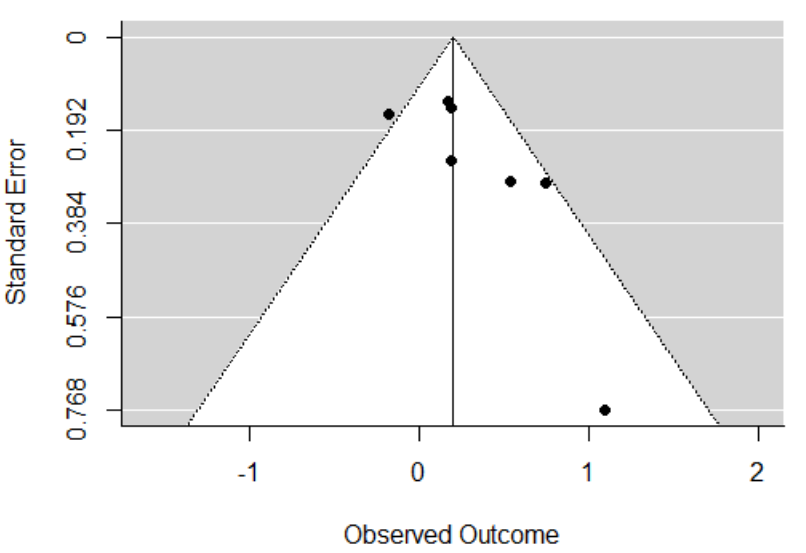


Case-control studies


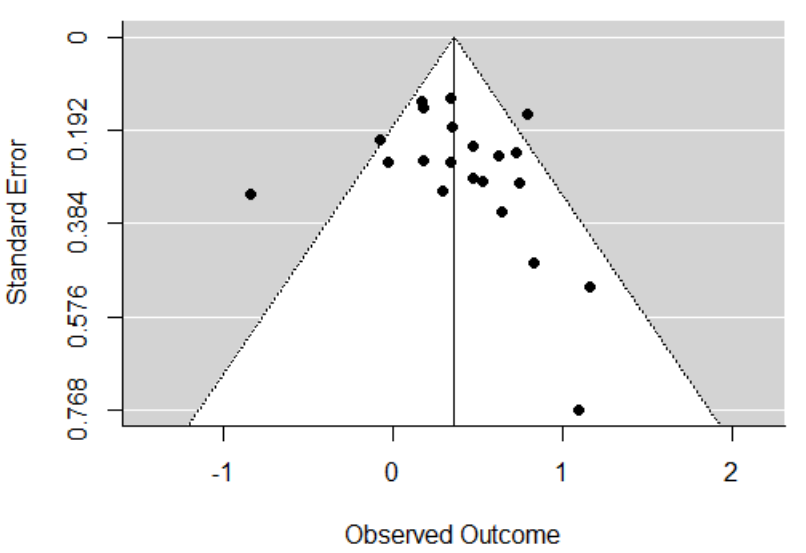


Cohort studies


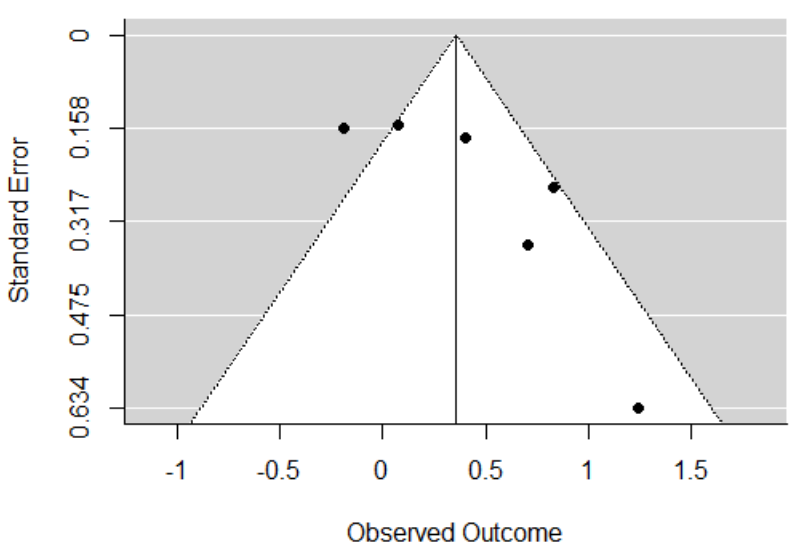


**Smoking - ML**

Overall


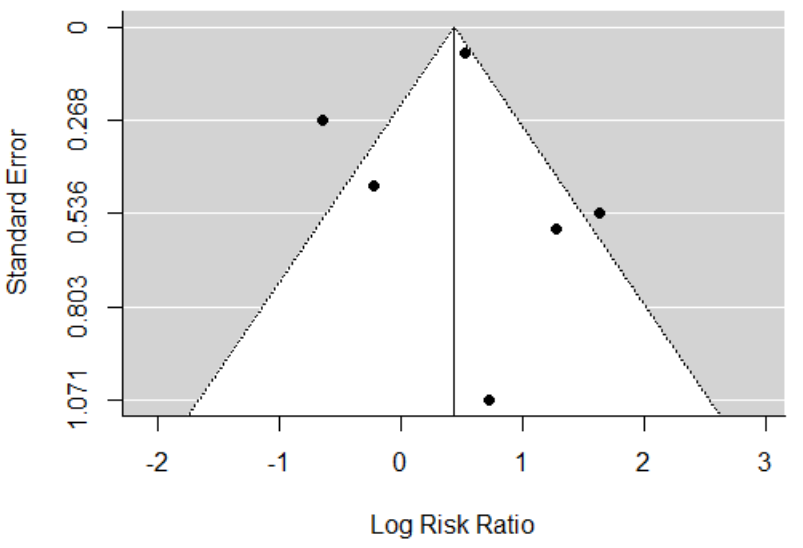


Current Smoker


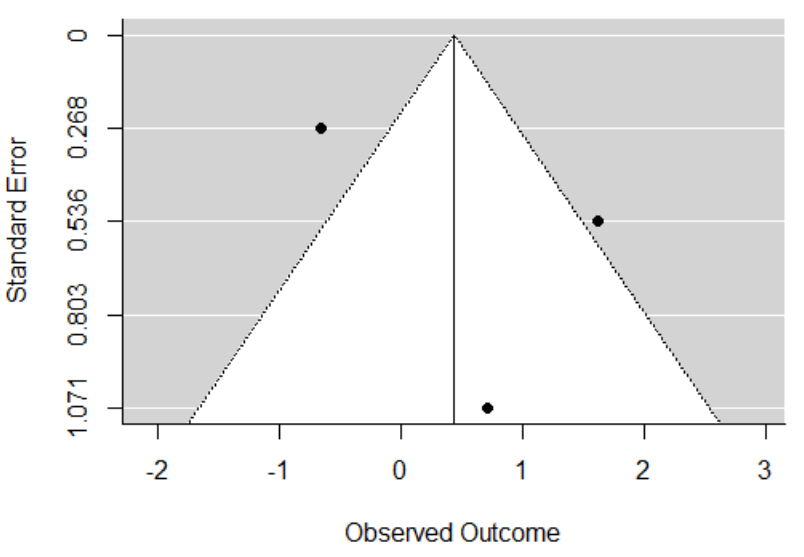


Ever Smoker


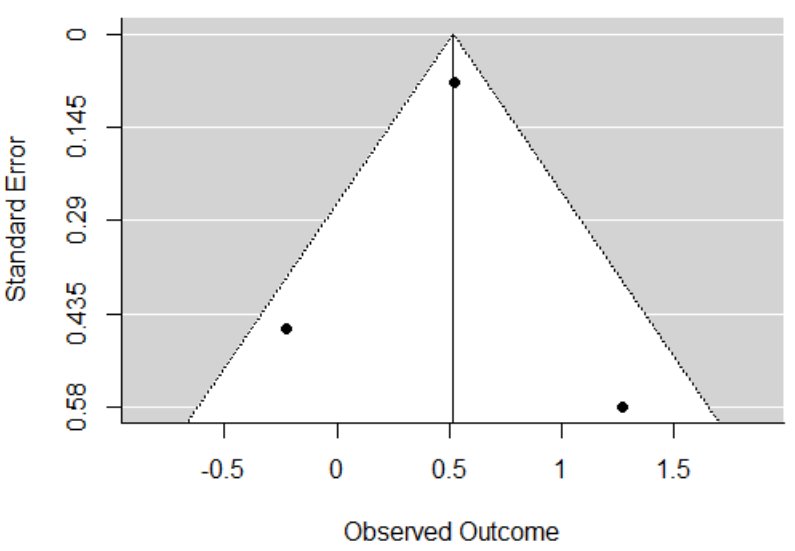


Case-control studies


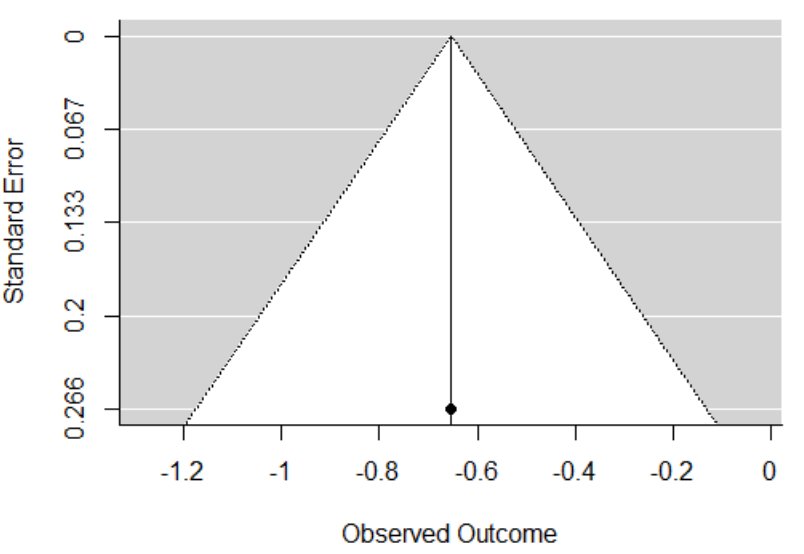


Cohort studies


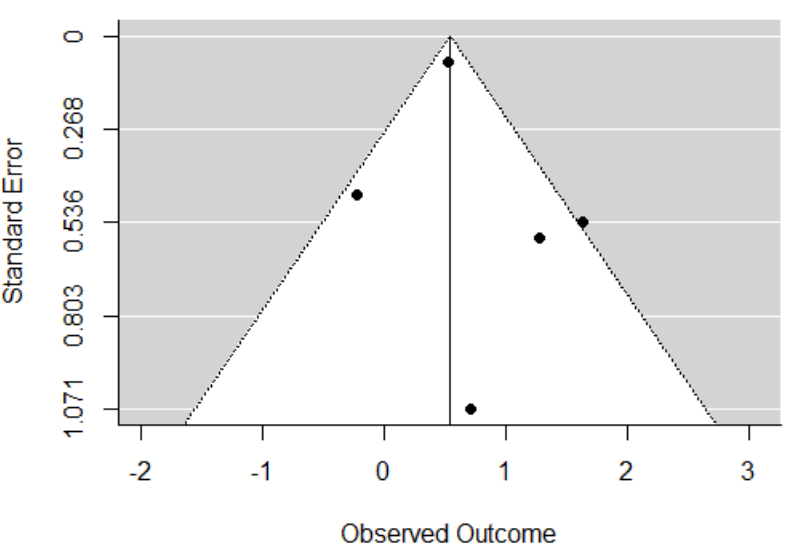


**Smoking - MDS**

Overall


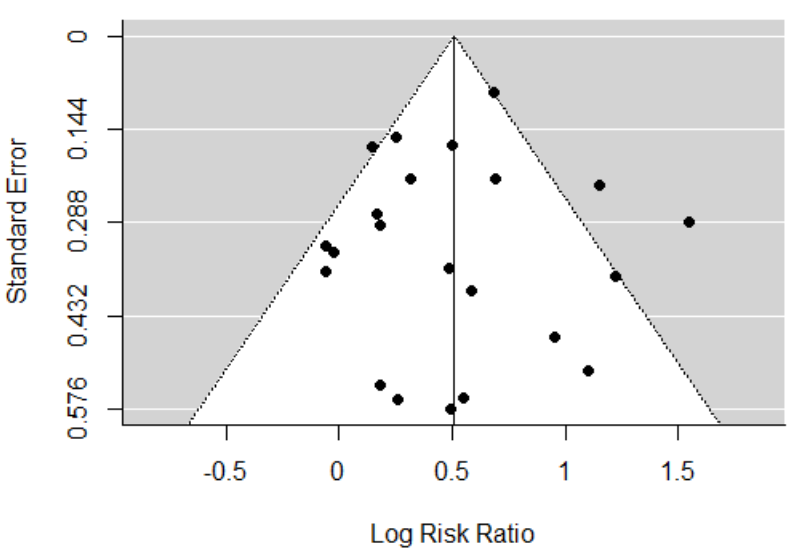


Current Smoker


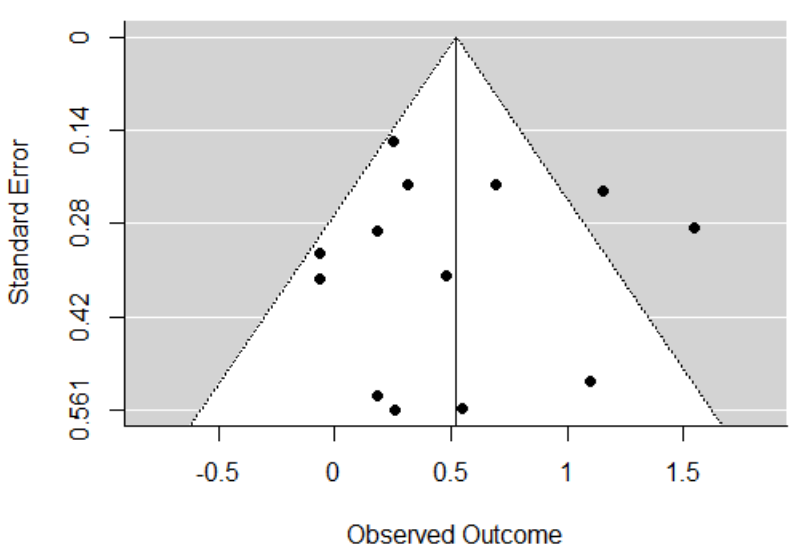


Ever Smoker


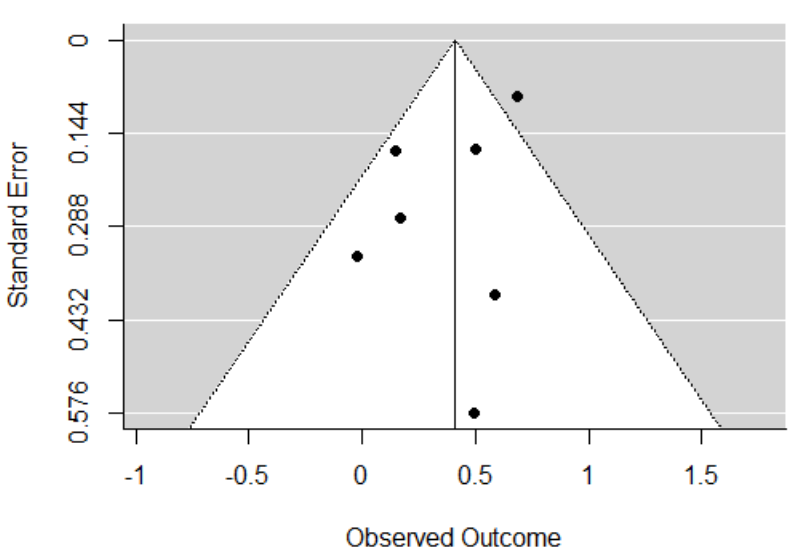


Case-control studies


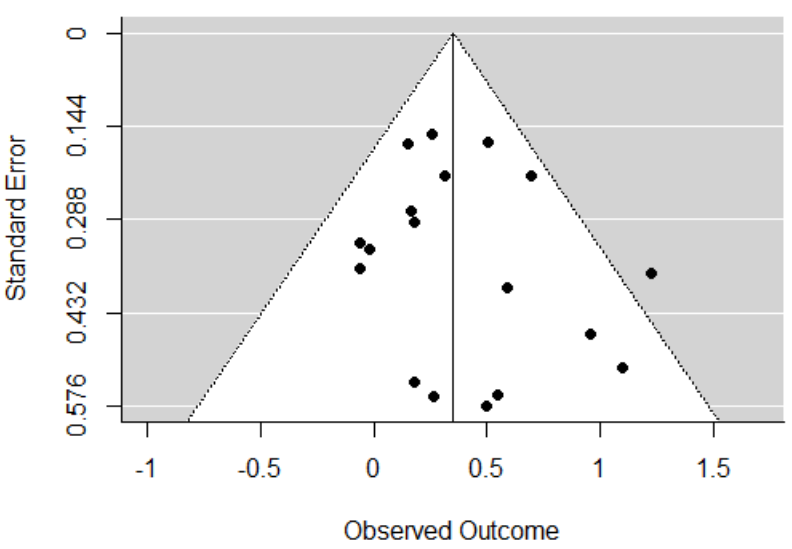


Cohort studies


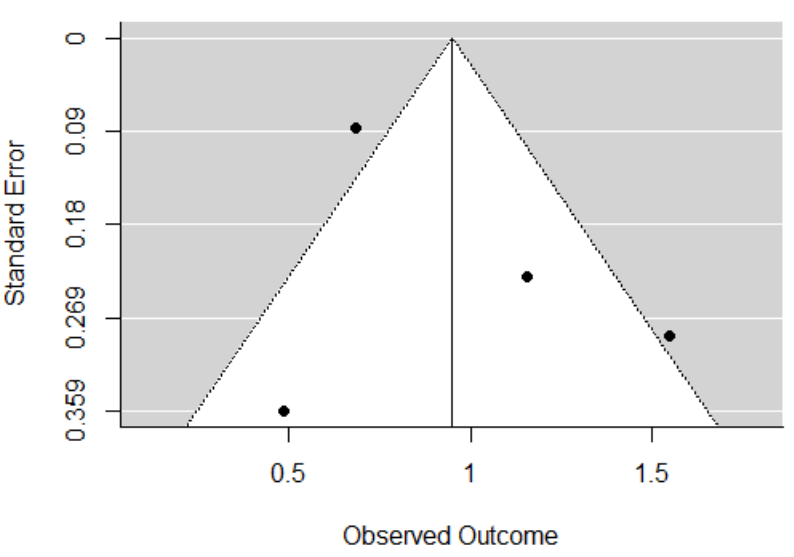


**Smoking - MPN**

Overall


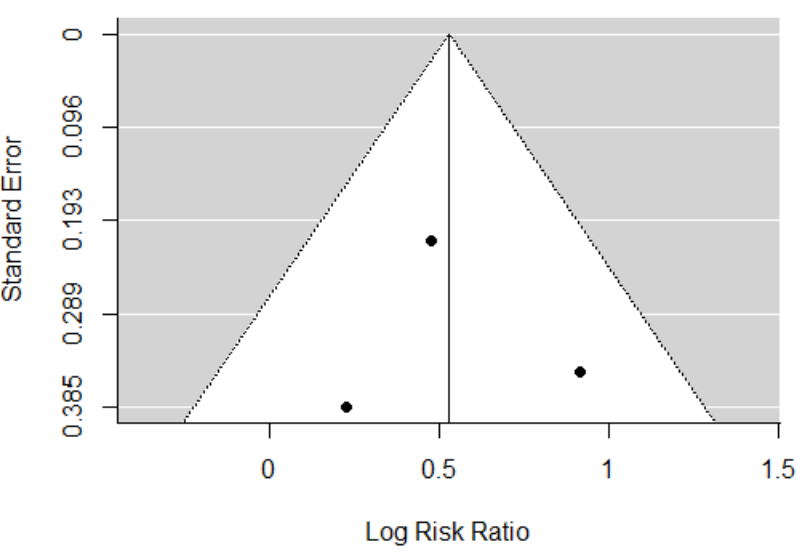


Current Smoker


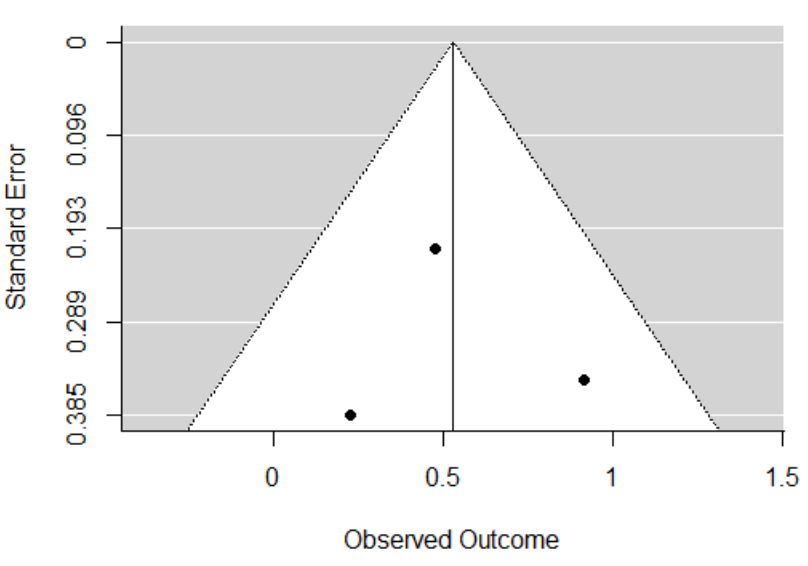


Case-control studies


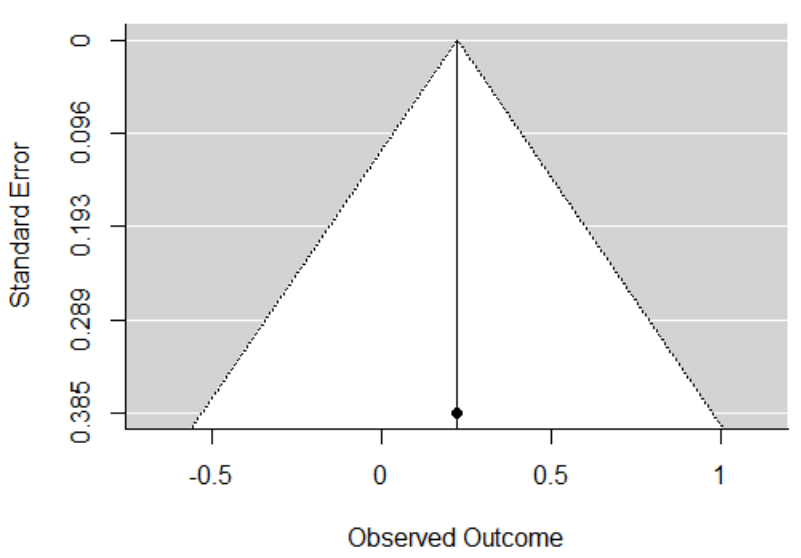


Cohort studies


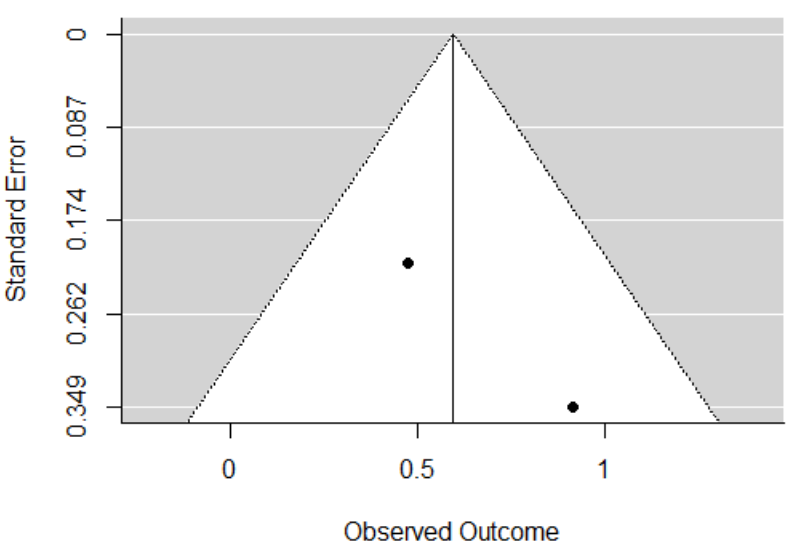

Supplement: Supplementary file 1 — Additional file 1. [file 12885_2021_7908_MOESM1_ESM.docx]
